# Fine-tuned characterization of *Staphylococcus aureus* Newbould 305, a strain associated with mild and chronic mastitis in bovines

**DOI:** 10.1186/s13567-014-0106-7

**Published:** 2014-10-14

**Authors:** Vincent Peton, Damien S Bouchard, Sintia Almeida, Lucie Rault, Hélène Falentin, Julien Jardin, Gwénaël Jan, David Hernandez, Patrice François, Jacques Schrenzel, Vasco Azevedo, Anderson Miyoshi, Nadia Berkova, Sergine Even, Yves Le Loir

**Affiliations:** INRA, UMR 1253 STLO, 65 rue de Saint Brieuc, 35042 Rennes Cedex, France; Agrocampus Ouest, UMR1253 STLO, 85 rue de Saint Brieuc, 35042 Rennes Cedex, France; Instituto de Ciências Biológicas, Universidade Federal de Minas Gerais, Belo Horizonte, MG Brasil; Genomic Research Laboratory, Service of Infectious Diseases, University of Geneva Hospitals (HUG), CH-1211 Geneva 14, Switzerland

## Abstract

*S. aureus* is a major aetiological agent of ruminant mastitis worldwide. The chronic nature of *S. aureus* mastitis makes it difficult to cure and prone to resurgence. In order to identify the bacterial factors involved in this chronicity, Newbould 305 (N305), a strain that can reproducibly induce mild and chronic mastitis in an experimental setting, was characterized in depth. We employed genomic and proteomic techniques combined with phenotype characterization, in order to comprehensively analyse N305. The results were compared with data obtained on *S. aureus* RF122, a strain representative of the major clone involved in severe bovine mastitis worldwide. Five mobile genetic elements were identified in the N305 genome as carrying virulence factors which correlated with phenotypic features such as cytotoxicity, mammary epithelial cell invasion or host-adaptation. In particular, the presence and characteristics of surface exposed proteins correlated well with the greater adhesion and internalization capacities of N305 in bovine mammary epithelial cells. N305 also displayed less diversity of toxin genes but secreted larger quantities of these toxins, associated with a higher cytotoxicity potential. Our data are consistent with the invasiveness and host-adaptation features which contribute to the chronicity of *S. aureus* mastitis. Mobile genetic elements, exoproteins and surface exposed proteins constitute good targets for further research to explore the underlying mechanisms related to mastitis chronicity.

**Electronic supplementary material:**

The online version of this article (doi:10.1186/s13567-014-0106-7) contains supplementary material, which is available to authorized users.

## Introduction

Mastitis is an inflammation of the mammary gland, which commonly results from a bacterial infection. This infection first induces local benign symptoms and can rapidly evolve towards general and severe symptoms and result in systemic infection. Mastitis dramatically impacts animal health and milk quality, and causes considerable economic losses throughout the global milk production chain [1]. *Staphylococcus aureus* is a major aetiological agent of ruminant mastitis, which is often difficult to cure and is prone to resurgence and chronicity [2]. There is therefore a need to better understand the mechanisms underlying the chronicity phenomenon in order to efficiently tackle and prevent *S. aureus* mastitis. Unlike *Escherichia coli* mastitis, the severity of which is mainly determined by host factors and not strain features, the severity of *S. aureus* mastitis mostly derives from inter-strain variations in terms of virulence potential [3,4]. Indeed, some *S. aureus* strains have reportedly induced chronic mastitis associated with mild symptoms, while others can cause severe mastitis.

Several hypotheses have been put forward to explain the chronicity of *S. aureus* mastitis, amongst which cytotoxicity, biofilm formation and tissue invasion have been the most widely investigated. *S. aureus* strains that secrete high levels of Panton-Valentine leukocidin and alpha toxin are associated with severe but not persistent mastitis [5-7]. By contrast, low-level cytotoxicity may facilitate persistence of the infection. Biofilm formation may also help *S. aureus* to resist antibiotic therapy and host defenses. The genetic loci *bap* and *ica*, which are involved in biofilm formation, are indeed associated with strains that cause less severe but more persistent mastitis [8]. Finally, the ability to invade and survive within mammary epithelial cells may also enable *S. aureus* to evade the host immune response and the curative treatments. All the evasive tactics that have been developed by *S. aureus* probably influence the rate of cures achieved by antibiotic therapies, and thus make *S. aureus* mastitis an infection difficult to treat.

In contrast with the huge efforts dedicated to the development of preventive and treatment strategies against *S. aureus* mastitis, little is still known about the genomics of *S. aureus* ruminant isolates. Compared to the hundreds of fully sequenced human isolates, only one bovine strain (RF122) and one ovine strain (ED133) have been fully sequenced, the former in 2007 [9] and the latter in 2010 [10]. We recently completed the set of publicly available genomic data on ruminant isolates through the sequencing and characterization of two ovine strains [3,11] and the recent release of three bovine *S. aureus* S1 [12], M186 [13], Newbould 305 (N305) genomes [14].

N305, a strain isolated in 1958 from a clinical case of cow mastitis in Orangeville, Ontario, Canada [15] has been used as a model strain of *S. aureus* mastitis isolates during numerous studies relative to vaccine development [16,17], antibiotic treatments [18], in vitro characterization [19] or in vivo experiments on bovine [20] and mouse models [21]. In particular, N305 was shown to reproducibly induce mild and chronic mastitis in the context of experimental infections [22,23]. But despite its widespread use as a prototype strain for chronic infections during academic and industrial research projects, N305 remains poorly characterized. A clearer understanding of how a strain is able to induce chronic mastitis is crucial to finding the Achilles heel in the pathogenicity of *S. aureus* and then developing more effective control strategies. In a previous work, we showed that *S. aureus* N305 and RF122 displayed different capacities for adhesion and internalization in bovine mammary epithelial cells [24]. To gain further insight into the characteristics of *S. aureus* that correlate with the hypotheses evoked to explain the chronicity of mastitis, we have now characterized N305 in depth at the genome, proteome and phenotype levels, and compared our findings with those obtained on *S. aureus* RF122. The results revealed that several features of N305 may contribute to the development of chronic mastitis, including mobile genetic elements (MGEs) and the types and production levels of toxins and surface proteins.

## Materials and methods

### Bacterial strains, growth conditions

*Staphylococcus aureus* Newbould 305 (hereinafter referred to as N305) [15] and RF122 [9] were isolated from cases of bovine mastitis. These strains are well characterized and can reproducibly induce severe mastitis (RF122; [25]; JR. Fitzgerald, University of Edinburgh, personal communication) or mild mastitis (N305) under experimental conditions [9,15]. N305 was kindly provided by Dr F. Gilbert (INRA Tours) and received as a lyophilized vial provided by D.S. Postle (College of Veterinary Medicine, Cornell University, Ithaca, New York) in 1975 and prepared from the original Newbould 305 isolate supplied by F.H.S. Newbould. One can therefore consider it is close to the original isolate (F. Gilbert and P. Rainard, INRA Tours, personal communication). Subcultures prior to invasion assays were performed overnight as follows. *S. aureus* strains were grown in brain heart infusion medium (BHI; Becton Dickinson, Le Pont de Claix, France) at 37 °C under agitation (180 rpm). The cultures were washed once with phosphate-buffered saline (PBS) and suspended at different concentrations in Dulbecco’s modified Eagle’s medium (DMEM; pH 7.4; D. Dutscher, Brumath, France). Bacterial concentrations in subcultures were estimated by spectrophotometric measurements at 600 nm (OD_600_). They were further confirmed using a micromethod, as previously described [26]. The resulting *S. aureus* populations (in CFU/mL) were determined on mannitol salt agar (MSA; D. Dutscher, Brumath, France) after 24 h of incubation at 37 °C.

### Genome comparison of RF122 and N305

The N305 strain was fully sequenced using the Illumina technique. The whole genome sequencing and assembly strategies are described in Bouchard et al. [14]. The N305 genome sequence was analysed using SurfG + to predict protein locations (potentially surface exposed (PSE), secreted, membrane and cytoplasmic proteins) [27], and PIPS, a software suite designed for the prediction of pathogenicity islands which, in an integrative manner, utilizes multiple features (such as atypical G + C content, codon usage deviation, virulence factors, hypothetical proteins, transposases, flanking tRNA and the absence of this structure in non-pathogenic organisms) to detect pathogenicity islands [28]. For the PIPS analysis, the genome sequences of the strains *Staphylococcus xylosus* C2a (kindly provided by R. Talon and S. Leroy, INRA Clermont-Ferrand) and *Staphylococcus carnosus* TM300 [29] were used as references. The assignment of protein function to the coding sequences (CDSs) of the two genomes (N305 and RF122) was performed manually using the results from BLASTP and the COG (Clusters of Orthologous Groups) [30].

These genome sequences are available at DDBJ/EMBL/GenBank under the accession numbers AKYW00000000 (N305) and NC_007622 (RF122).

### Phenotype characterization

#### Biofilm formation

The two *S. aureus* strains were assayed for biofilm formation using crystal violet staining. The bacteria were subcultured twice in BHI before adjusting the OD_600nm_ to 0.004 (corresponding approximately to 10^6^ CFU/mL) in BHI containing 4 g/L glucose (hereinafter named BHIglu), in order to promote biofilm formation. Growth was performed in 96-well microtiter plates with 200 μL of bacterial suspension per well. BHIglu alone was used as a negative control. The biofilm staining assays were performed following an incubation period of 24 h at 37 °C. The microtiter plates were washed twice with phosphate-buffered saline (PBS), fixed for 20 min at 80 °C and stained for 10 min with 1% (w/v) crystal violet solution, freshly diluted 10-fold in distilled water. The plates were then washed twice with distilled water and allowed to dry at room temperature. The crystal violet was dissolved in 200 μL of acetic acid solution (33% in distilled water) for 10 min under gentle agitation. Optical density was measured at 595 nm using a Spectramax spectrometer. The *S. aureus* strain MW2 was used as an internal normalization standard and biofilm formation by N305 and RF122 was expressed as a ratio OD_N305_/OD_MW2_, and OD_RF122_/OD_MW2_.

#### Cytotoxicity

To assess their cytotoxic effect, the viability of MAC-T cells was measured during their incubation with a culture supernatant at 24 h post-infection using methylthiazolyldiphenyltetrazolium bromide (MTT), as previously described [31]. Briefly, the strains were pre-cultured in RPMI 1640 (Sigma, Saint Quentin Fallavier, France) supplemented with 2.2′dipyridyl (200 mM, Sigma) and then diluted 1000-fold in fresh RPMI + deferoxamine mesylate (152 mM, Sigma). These iron-depleted conditions were previously shown to mimic the mastitis context and to increase the expression of virulence factors [32]. *S. aureus* strains were grown without agitation at 37 °C under microaerophilic conditions, in 50 mL tubes. After 24 h of growth, the cultures were centrifuged and the supernatants were filtered on 0.22 μm units before being diluted in fresh DMEM at a ratio of 1:1. The cells were incubated for 24 h in this medium and then 0.5 mg/mL MTT were added for 4 h at 37 °C in 5% CO_2_. The medium was removed and isopropanol was added for 30 min under shaking at 350 rpm. Absorbance was read at 570 nm with a background at 690 nm. Cells treated with PBS diluted in DMEM (ratio 1:1) were used as a negative control (100% viability). Relative viability was expressed versus PBS-treated cells.

#### Proteolysis

Proteolysis capacity of *S. aureus* strains was assessed using a simple plate assay to visualize milk protein degradation. Briefly, culture supernatants were prepared as described in cytotoxicity assays, filtered on 0.22 μm units, and concentrated 10x in a Speed Vac Concentrator (Savant, Thermo Scientific). Fifty μL of these supernatants were deposited in wells in PCA agar medium supplemented with 5% of skimmed milk. A translucent halo around the wells indicates the casein lysis.

#### Plasma coagulation

To confirm the presence in N305 of a bovine variant of von Willebrand binding protein, plasma samples from cows and goats were prepared from blood collected in EDTA-coated tubes (blood samples kindly provided by Jacques Lassalas, UMR1348 PEGASE, INRA Agrocampus Ouest, Rennes, France). Rabbit plasma (Sigma) was used as a positive control for coagulase activity. A volume corresponding to 10^8^ CFU of N305 or RF122 (overnight culture on BHI) was pelleted, washed once in PBS and re-suspended in 300 μL serum. Coagulation was checked after 4 h of incubation at 37 °C by inverting the tubes.

### 2-Dimensional gel electrophoresis for the total proteome and secretome of RF122 and N305

For 2D-PAGE, protein samples were prepared as described previously, with some minor changes [3]. Briefly, *S. aureus* strains were pre-cultured in RPMI 1640 supplemented with 2.2′-dipyridyl (200 μM) to chelate extracellular iron and consequently reduce intracellular iron stocks. The cultures were then diluted 1:1000 in fresh RPMI 1640 with deferoxamine (152 μM). *S. aureus* strains were grown without agitation at 37 °C under microaerophilic conditions, in 500 mL flasks for the supernatant fraction or in 50 mL tubes for the total fraction and shaving experiments. These conditions were previously shown to best mimic growth in vivo during mastitis [32]. The cultures were centrifuged at 7000 *g* for 10 min and the supernatants were filtered through a 0.22 μm filter unit. Total cell lysate proteins were obtained from bacterial pellets, as previously described [32].The supernatant proteins were precipitated with 10% TCA at 4 °C overnight. The samples were centrifuged at 9000 *g* for 1 h at 4 °C. Protein pellets were washed three times with 96% ethanol and then dried. Proteins were solubilized in urea 8 M. The protein concentration was determined using a Bradford test (Sigma). A constant amount of proteins was used for each acrylamide gel so as to enable determination of a relative abundance of a given protein with respect to the total of secreted proteins. Three biological replicates were used for each analysis.

To purify the protein extracts, a 2D Clean Up kit (GE Healthcare, Orsay, France) was used according to the manufacturer’s instructions, and then the samples were treated as described previously [3]. Images of the gels were analysed using SameSpot software (TotalLab Ltd., Proteomics consult, Belgium), as previously described [1,32,33]. Spot volumes were determined and normalized with regard to the total volume of the gel. After this normalization, spots with a minimal fold change of 2 and an ANOVA e-value lower than 0.005 were selected for mass spectrometry analysis. The gel pieces were processed exactly as described previously [3,34]. A shaving technique was used to analyse the PSE proteins. *S. aureus* cultures were centrifuged at 7000 *g* for 10 min and the total cell and supernatant fractions were treated separately. The pellets were washed twice with PBS. The bacteria were re-suspended in PBS with sufficient 5 mM DTT to reach 20 units of OD_600nm_. Five hundred μL of this suspension were incubated with 20 μg trypsin for 1 h at 37 °C under agitation (180 rpm) and then centrifuged at 10 000 *g*. The supernatants were filtered through a 0.22 μm filter and incubated overnight with 1 μg trypsin at 37 °C under agitation. Trypsin digestion was stopped by adding 15 μL 5% TFA. The controls did not display any significant loss of viability from the counts of CFUs before and after shaving.

### Identification of proteins

Proteins were identified using NanoLC-ESI-MS/MS as described by Le Maréchal et al*.* [1] with some minor modifications. Peptides were identified using the X! Tandem software. The database used for protein identification was composed of predicted proteins based on the RF122 and N305 genome sequences [9,14], together with protein data on *Staphylococcus aureus* (taxon 1280) in the UniProtKB database [35], in order to obtain a statistically significant identification. To achieve valid identification with a high degree of confidence, each protein must have a minimum of two peptides corresponding to a *p*-value lower than 0.05. An auto-validation of peptides from the X!Tandem search results was performed using X!TandemPipeline [36]. Prediction of the sub-cellular localization of proteins was achieved using PSORTb 3.0.2 software [37]. Validated and identified proteins were then sorted by Clusters of Orthologous Groups (COGs) using EggNOG 3.0 software [38].

Regarding proteins identified by trypsin shaving, only those identified with at least two peptides in one strain and less than two peptides in the other strain were considered as more abundant in the first strain.

### Statistical analysis

All experiments were carried out in triplicates (biological repeats). The differences in biofilm formation and cytotoxicity assays were assessed using paired Student’s *t* tests considering a *P* value lower than 0.05. In proteome analyses, proteins were considered as over expressed in total proteome or exoproteome when ANOVA e-value given by SameSpot software was lower than 0.005 and the minimal fold change was 2.

## Results

### Comparison of the gene contents of *S. aureus* RF122 and Newbould 305

The general features of the N305 genome are summarized in Table 1, which also includes the general features of RF122 [9]. Predicted proteins were functionally categorized using the COGs database. The COGs distributions were similar in the two genomes (see Additional file 1). The majority of the genes (*n* = 2518) were common to both strains. The core genome covers up to 91.5% and 94.5% of the CDSs of N305 and RF122, respectively. However, a comparison of the sequences of N305 and RF122 revealed some differences in their overall genome content. A total of 48 371 single nucleotide polymorphisms (SNPs) were found in N305 when compared to RF122. Among the SNPs located in the CDSs, 10 470 were non-synonymous and 1690 corresponded to insertions or deletions. In order to better understand the genomic features responsible for the phenotypic differences between N305 and RF122, we further analysed the gene content of *S. aureus* N305 in terms of putative MGE, which appeared to be the principal source of variability between the two genomes.

**Table 1:** **General features of Newbould 305 and comparison to RF122 genome**

	**Newbould 305**	**RF122**
Size (bp)	2 791 699	2 742 531
GC %	32.8	32.8
Number of CDSs	2752	2664
Number of SNPs	48 371	
CORE N305 x RF122	2518 (e-value = 1^e-5^/identity > 70%)	
Pseudogenes	6	76
Protein coding genes with COGs	2052 (70.53%)	1903 (70.74%)
rRNA genes	5S rRNA = 7	5S rRNA = 6
	16S rRNA = 6	16S rRNA = 5
	23S rRNA = 7	23S rRNA = 5
tRNA genes	56	64

**Figure 1:**
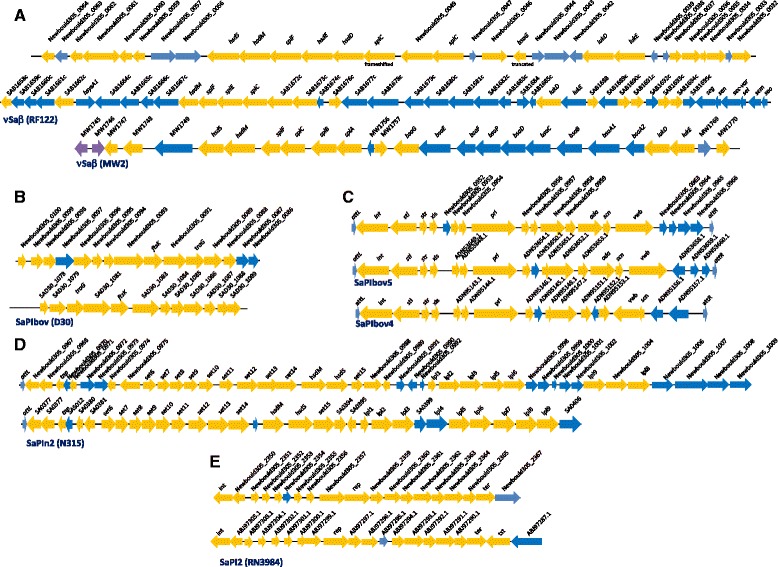
**Putative SaPIs found in the Newbould 305 genome.** Putative SaPIs of N305 are presented in the upper lines, and compared with the most closely related SaPI found in public databases. **A**. Comparison of the pathogenicity island SaPIN305_1 with νSaβ in the RF122 and MW2 strains. **B**. Comparison of the pathogenicity island SaPIN305_2 with SaPIbov found in the *S. aureus* D30 strain [39]. **C**. Comparison of the pathogenicity island SaPIN305_3 with SaPIbov5 and SaPIbov4. **D**. Comparison of the pathogenicity island SaPIN305_4 with SaPIn2 found in the *S. aureus* strain N315 [40]. **E**. Comparison of the pathogenicity island SaPIN305_5 with SaPI2 found in *S. aureus* RN3984 [41]. Arrows represent open reading frames and their orientations. Orange: common genes shared with the most closely related SaPI represented on the Figure; blue: additional or different genes.

Analysis of the N305 genome using the PIPS pipeline [28] predicted the presence of five putative *S. aureus* Pathogenicity Islands (SaPIs). SaPI-N305_1 (contig 1) contained 35 predicted CDSs markedly similar to the CDSs of νSaβ already described in RF122 and *S. aureus* MW2 (Figure 1A). SaPI-N305_2 (contig 1) contained 15 CDSs markedly similar to a SaPIbov found in strain D30 (isolated in the context of human nasal carriage) [39] (Figure 1B). SaPI-N305_3 and SaPI-N305_4 were two adjacent putative SaPIs (contig 2) containing 19 and 28 CDSs, respectively. SaPI-N305_3 shared marked similarity with SaPIbov4 and SaPIbov5, which both carry a bovine variant of von Willebrand factor binding protein (*vwb*^*Sbo5*^) (Figure 1C). SaPI-N305_4 shared similarity with SaPIn2, which was first described in the human *S. aureus* strain, N315 [40] (Figure 1D). SaPI-N305_5 (contig 6) contained 35 CDSs, 17 of which were found to be very similar to SaPI2. PIPS analysis also revealed the presence of two putative bacteriophages, on contig 5, containing 34 CDSs and 19 CDSs with homology to Bacteriophage 80alpha and bacteriophage 187, respectively.

We further analysed the N305 genome while focusing on genes suspected of being implicated in the chronicity of infections, i.e. genes reportedly involved in biofilm formation, cytotoxicity and invasiveness (Table 2). This revealed differences between the RF122 and N305 strains. Some genes were only found in N305, encoding proteins such as the delta-hemolysin (Hld), the bovine variant of the von Willebrand binding protein (vWb^Sbo5^), fibronectin binding protein B (FnbB), *S. aureus* surface protein G (SasG) and 1-acyl-sn-glycerol-3-phosphate acyltransferase (PlsC). These factors are involved in the colonization of host tissue (vWb^Sbo5^, FnbB, SasG, and PlsC) or cytotoxic effects (Hld). A cluster of *set* genes (staphylococcal enterotoxins) was also found in SaPI-N305_4 (Figure 1D and Table 2). None of the strains possess the *bap* gene, which encodes a protein implicated in biofilm synthesis in *S. aureus*. However, they both have the *icaADBC* operon and the *icaR* regulator. In terms of virulence regulation, the accessory gene regulator (*agr* system) of N305 is of type I (while RF122 is *agr*II), as shown by the genome sequence analysis.

**Table 2:** **Presence (+) or absence (-) of virulence-associated genes in RF122 and Newbould 305**

**Gene name**			
**Biofilm formation**	**Function**	**NB305**	**RF122**
*ica* operon	Biofilm synthesis *ica* operon	+	+
*bap*	Biofilm associated protein	-	-
*aap*	Accumulation-associated protein (associated to biofilm formation)	+*	-
*ypfP, ltaA, ltaS, tag* and *dlt* operons	Teichoic and Lipoteichoic acid synthesis	+	+
*cap* operon	Capsular polysaccharide biosynthesis	+	+
**Secreted toxins**	**Function**	**NB305**	**RF122**
*lukE*	Leukocidin LukE precursor	+	+
*lukD*	Leukocidin LukD precursor	+	+
*lukM*	Leukocidin chain lukM precursor	-	+
*lukF’-PV*	Panton-Valentine leukocidin LukF’-PV chain	-	+
*lukF*	Leukocidin F subunit	+	+
*lukS*	leukocidin/hemolysin toxin subunit S	+	+
*hla*	Alpha-hemolysin precursor	+	+
*hlb*	Beta-hemolysin precursor	+	+
*hlg (subunits A, B, C)*	Gamma-hemolysin component	+	+
*hld*	Delta-hemolysin	+	-
*atl*	Bifunctional autolysin precursor	+	+
*tst*	Toxic shock syndrome toxin 1	-	+
*eta*	Exfoliative A	+	+
*etb*	Exfoliative B	-	+
*sea*	Staphylococcal enterotoxin A	+	+
*sec bov*	Bovine variant of Staphylococcal enterotoxin C	-	+
*seg*	Staphylococcal enterotoxin G	-	+
*sei*	Staphylococcal enterotoxin I	-	+
*sel*	Staphylococcal enterotoxin L	-	+
*sen*	Staphylococcal enterotoxin N	-	+
*seo*	Staphylococcal enterotoxin O	-	+
**Colonisation factors**	**Function**	**NB305**	**RF122**
*vwb*	Secreted von Willebrand factor-binding protein (Wbp) precursor	+	+
*vwb* ^*Sbo5*^	SaPI-encoded variant of Wbp carried by SaPIbov5	+	-
*clfA*	Clumping factor A	+	+
*clfB*	Clumping factor B	+	+
*fnbA*	Fibronectin binding protein A	+	+
*fnbB*	Fibronectin binding protein B	+	-
*stl*	Transcriptional Repressor SaPI	+	+
*eap*	Extracellular adherence protein	+	+
*sasG*	*S. aureus* surface protein G	+	-
*plsC*	1-acyl-sn-glycerol-3-phosphate acyltransferase	+	-
*sak*	Staphylokinase	-	-
*spa*	Immunoglobulin G binding protein A precursor	+	+

^*^Newbould 305_2513 possess a CWA domain and is 67% similar to *aap* gene of *S. epidermidis* (GeneBank accession number YP_189945).

Some genes were only found in RF122, encoding for leukocidin M (LukM) and Panton-Valentin leukocidin F (LukF’-PV), toxic shock syndrome toxin 1 (TSST-1), exfoliative toxin B (Etb) and the enterotoxins Cbov/G/I/L/N/O (SECbov, SEG, SEI, SEL, SEN, SEO). All these proteins are secreted toxins.

**Figure 2:**
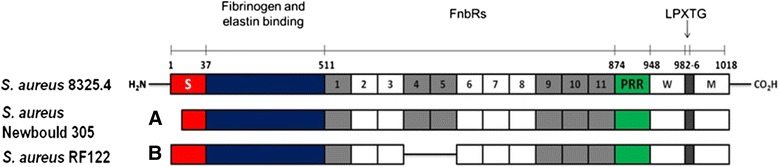
**Comparison of the FnbA found in RF122 and N305 with that of** ***S. aureus*** **8325.4** ***.*** The FnbA in *S. aureus* 8325.4 (SWISS-Prot P14738) contains 11 Fn-binding sites. FnbA from *S. aureus* Newbould 305 **(A)** and RF122 **(B)** showing the approximate positions of the predicted fibronectin binding regions (FnbRs). High-affinity FnbRs are shaded. Signal peptide (S) in red, proline-rich repeats (PRR) in green; cell wall-spanning sequence (W) in white; membrane-spanning region (M) in white.

Particular focus on microbial surface components recognizing adhesive matrix molecule (MSCRAMMs) genes revealed that their number was higher in N305 than in RF122. Although both strains carry the gene coding for fibronectin-binding protein A (*fnbA*), we found that the *fnbA* sequence in N305 had two additional fibronectin-binding domains when compared to that of RF122 (Figure 2).

### The same biofilm formation but broader coagulase activity spectrum and higher cytotoxic capacities for N305 during in vitro assays

**Figure 3:**
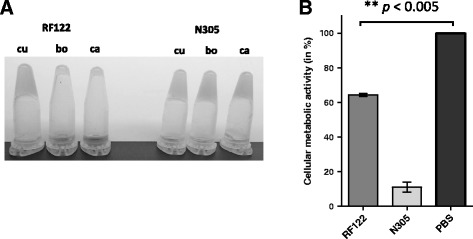
**Phenotypic characterization of** ***S. aureus*** **N305 and comparison with RF122. A**. Coagulase activity of RF122 and N305. A 10^8^ CFU aliquot was prepared from an overnight culture of each strain, washed and re-suspended in rabbit (cu), bovine (bo) or goat (ca) plasma. After a 4-h incubation at 37 °C, the level of coagulation of observed by tilting the tubes. **B**. Cytotoxic effects of *S. aureus* RF122 and N305 supernatants on MAC-T cells. *S. aureus* strains were grown for 24 h in RPMI with 50 mM deferoxamine. Supernatants (or PBS for the control condition) were mixed in DMEM at a ratio of 1:1 and incubated with MAC-T cells. Cytotoxicity was assessed using the MTT test and relative viability was expressed with regard to PBS-treated cells. Each experiment was performed in triplicate, and differences between the groups were compared using Student’s *t* test. **P < 0.005.

Based on the differences in gene content revealed by sequence analysis, phenotypic differences could be expected in terms of biofilm formation, coagulase activity, cytotoxicity and invasive capacities. These four properties were tested in vitro at the phenotype level (see Materials and methods for details). In line with the absence of differences in terms of the content of genes involved in biofilm formation, the two strains did not display any significant differences in phenotype. Indeed, RF122 and N305 revealed the same ability to form a biofilm on an abiotic surface, as determined by Crystal violet staining on a plastic surface (data not shown). By contrast, with respect to coagulase activity, the presence of *vwb*^*Sbo5*^ (Newbould 305_0962) in the N305 genome (Figure 1C), whose product was also found in the N305 surface exposed protein (Additional file 2), was clearly associated with an increased range of coagulase activity. N305 indeed clotted rabbit, goat and bovine plasma, whereas RF122 only clotted rabbit plasma (Figure 3A). Furthermore, N305 displayed greater cytotoxicity on MAC-T cells than RF122 after co-incubation with their respective supernatants. The viability of cells incubated with the N305 supernatant was 89% lower than under control conditions, whereas the viability of cells incubated with the RF122 supernatant was 36% lower (Figure 3B). N305 supernatant also presented a higher ability to hydrolyse caseins as shown by the translucent halo around the well of N305 deposit (Additional file 3).

### Comprehensive analysis of the proteome of *S. aureus* Newbould 305

**Figure 4:**
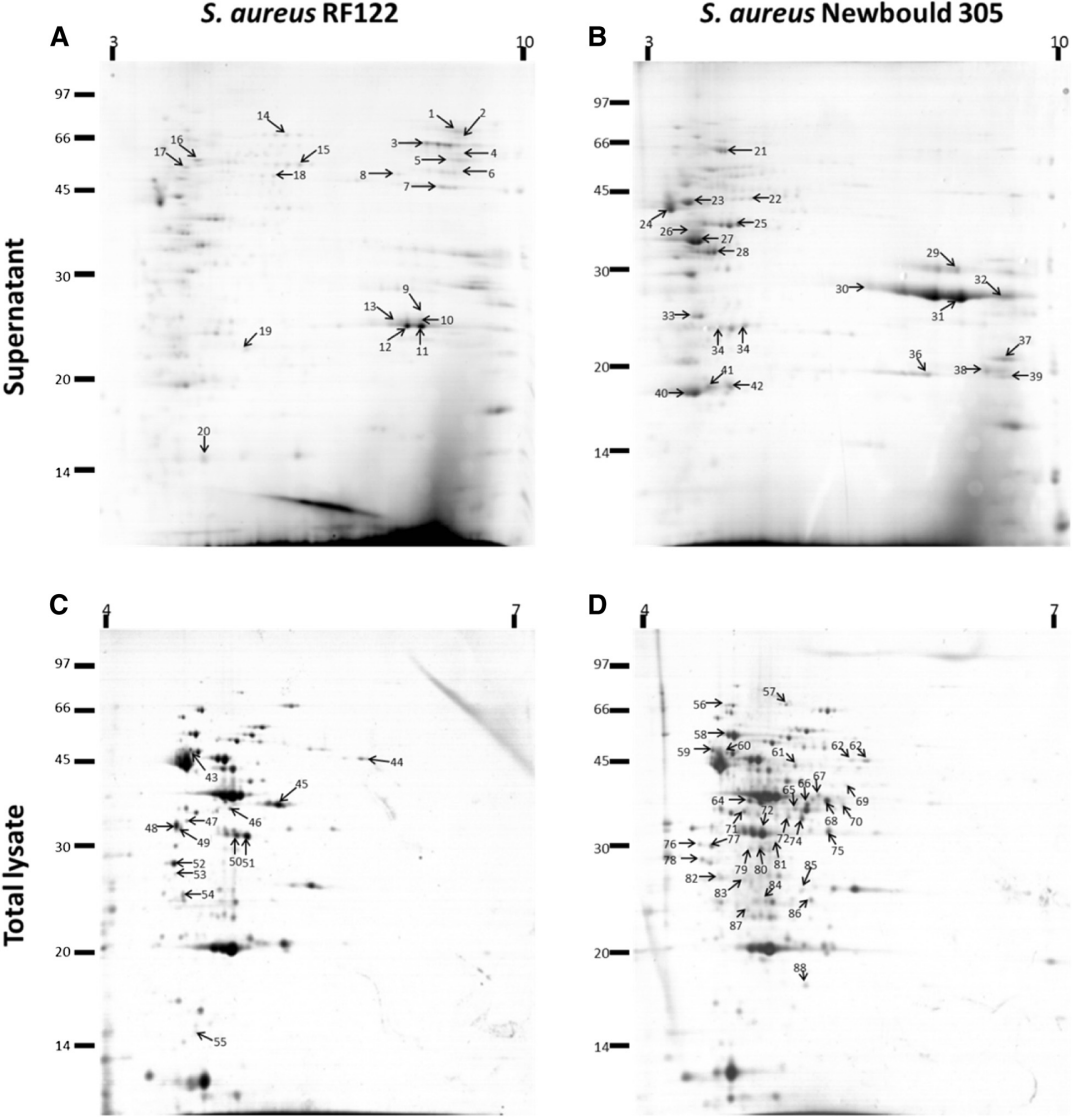
**2D PAGE of** ***S. aureus*** **RF122 and N305 protein extracts.** Supernatants **(A and B)** and total proteomes **(C and D)** were harvested during the stationary phase. Supernatant proteins were precipitated with 10% trichlororacetic acid and washed with high-grade ethanol. Samples were purified using the 2D Clean Up kit (GE Healthcare) then separated by isoelectrofocalization on pH4-7 strips (total proteome) or pH3-11NL strips (supernatant) for the first dimension, and by 12% polyacrylamide gels for the second dimension. The gels were stained with Biosafe (Biorad) Coomassie blue, according to the manufacturer’s instructions. Arrows indicate spots that were over-expressed.

A comprehensive analysis of the N305 proteome (total cell, surface and secreted proteins) was carried out on cultures grown under conditions mimicking the context of mastitis, as described previously [32]. The N305 proteome was compared with that of RF122. A total of 215 proteins were categorized as being differentially produced in N305 or RF122, and were further identified. Sixty-eight proteins (31.6%) were identified as being over-expressed by RF122, and 147 (68.4%) by N305. In more detail, a total of 33 proteins (15.4% of total proteins) were identified in the culture supernatant (Additional file 4), 11 proteins being more abundant in RF122 and 22 in N305. Supernatant gels (Figure 4A and B) displayed the most flagrant proteomic differences between N305 and RF122. In the total cell lysate, 36 proteins (16.7% of total proteins) were identified (Additional file 5), ten proteins being more abundant in RF122 and 26 in N305. Trypsin shaving enabled the identification of up to 409 proteins because of the sensitivity of this method, and included 146 proteins that were relatively more abundant in one strain (67.9% of total proteins) (Additional file 2), with 47 proteins more abundant in RF122 and 99 in N305.

The proteins were classified into COGs as a function of their annotation. Most of the secreted and PSE proteins identified here belonged to cellular processes and signalling categories or were poorly-characterized proteins. Among these, a number of exoproteases were found to be common to both strains (Additional files 4 and 2), while some exoproteases such as Spl proteases (SaPI-N305_1), were also specifically found in N305. Virulence factors (classified in the category of poorly-characterized proteins) were also found to be specific to N305. These included haemolysins and leukotoxins (alpha- and gamma-haemolysins, LukS/F) and proteins involved in adhesion to host tissues (e.g. Newbould305_1324, encoding an extracellular matrix and plasma binding protein) and in evasion of the host immune response (e.g. Spa, vWbp^Sbo5^). By contrast, RF122 was found to produce leukotoxins (e.g. LukM/F’ and LukE) and enterotoxins (e.g. bovine variant of the staphylococcal enterotoxin C *sec-bov*) as well as iron metabolism proteins (e.g. IsdB and D). As expected, total proteins (as well as the surface exposed proteins identified by trypsin shaving) comprised many more proteins that those grouped in the Metabolism categories.

## Discussion

The outcomes of *S. aureus* mastitis can vary markedly, and are mostly linked to strain-dependent features [3]. By contrast with mastitis induced by other pathogens, one of the most problematic traits of *S. aureus* mastitis is its low cure rate and its propensity to chronicity. Here, we have finely characterized N305 [15], an *S. aureus* strain that reproducibly induces mild and chronic mastitis in an experimental cow model, and compared it to RF122, a well-documented and highly virulent *S. aureus* strain [25] (J. Ross Fitzgerald, personal communication) which is representative of a common clone which frequently causes bovine mastitis [9,15]. These two strains belong to distinct clonal complexes (CC97/ST115 for N305, and CC133/ST151 for RF122), with both strains clustering in groups that include several other bovine strains and strains of human or non-specified origin. Although RF122 has been described as an archetypal bovine strain, it is closely related to human strains. By contrast, N305 is strongly associated with a bovine host [42,43].

Despite their phylogenetic divergence, a comparison of their genome sequences revealed some strong similarities, with a core genome (set of genes common to N305 and RF122) comprising more than 91% of their gene content (Table 1). However, certain genomic differences were found in their pseudogene contents. The RF122 genome reportedly contains 70 pseudogenes [9], whereas only six pseudogenes are predicted in the N305 genome. Significant differences were also found regarding the genes encoding toxins and surface proteins involved in host invasion. These latter genomic features were confirmed by phenotypic and proteomic characterizations.

In terms of gene regulation, we found that N305 is *agr*I, while RF122 is *agr*II. The *agr* system plays a central role in *S. aureus* virulence expression. Interestingly, *agr*I bovine strains were recently shown associated to persistence, with high intracellular survival and probably a better adaptation to an intracellular niche, whereas *agr*II was associated with a low intracellular survival and a probable extracellular niche [44].

### An inventory of potential mastitis-associated functions in N305 reflects host adaptation and propensity to chronicity

A comparison between the N305 and RF122 genomes revealed differences regarding the genes encoding PSE and secreted proteins. The complete *spl* operon (*splA, B, C, D, E, F*; although *splC* is pseudogenized) was found in N305 whereas only *splB*, *splC, splE*, and *splF* were found in the RF122 genome sequence. In addition, a series of four serine proteases, including SplB (Newbould 305_0049), SplE, D, and F, encoded by the *spl* operon, was found to be relatively more abundant in the N305 supernatant (Table 3). In agreement, we observed a higher ability of N305 to degrade caseins, which is associated to SplA, SplB and SplC in *S. aureus* [45,46]. Other differences were found in toxin gene contents, which correlated with differences in the composition of MGEs. RF122, which is associated with severe mastitis, was found to be well-equipped with genes encoding enterotoxins, haemolysins, exfoliatin, toxic shock syndrome toxin, and leukocidin, whereas the N305 genome carried fewer and different toxin genes. *S. aureus* RF122 carried numerous enterotoxin genes, including the bovine-associated variant of staphylococcal enterotoxin C which is involved in disturbances affecting the immune response and is carried by SaPIbov1 [9]. Unlike N305, RF122 was found to possess *luk*M and *luk*F’-PV (hereinafter referred to as *luk*F’), a *luk*F-PV variant carried by the prophage φPV83-pro [47]. These genes form LukM/F’ which is involved in cytotoxicity against polynuclear neutrophils, mainly described during a strong inflammatory reaction in the mammary gland. LukM/F’ is more efficient against bovine leukocytes than any other staphylococcal toxin, including Panton-Valentine leukocidin [5,48,49]. As for other leukocidins, both strains contained the *luk*D/E genes and *luk*F/S genes, also described as *luk*B/A or *luk*G/H, respectively [50,51], and LukS and LukF subunits were only found in N305 protein samples (Table 3). However, the LukM/F’ leukotoxin, found in RF122, has a higher affinity for bovine myeloid lineage cells [5,52] and has been shown to be associated with severe clinical mastitis and to be highly prevalent in gangrenous mastitis isolates [7,53]. Other toxin genes are absent from the N305 genome when compared to that of RF122, which might explain its less virulent phenotype during mastitis. Genes encoding for toxic shock syndrome toxin, exfoliatin B and enterotoxins, such as *sec* which is carried by SaPIbov, and *seg*, *sei*, *sen*, *seo*, carried by an enterotoxin gene cluster (*egc*), were indeed present in RF122 and absent from the N305 genome. Enterotoxins play a major role during staphylococcal infections and can induce a strong immune response in the mammary gland [54]. Analysis of the secretomes of RF122 and N305 confirmed some of the differences observed in the gene content, but it also revealed differences in the production of virulence factors whose genes were present in both genomes. Hence the production of haemolysins (α- and γ-haemolysin) was more pronounced in N305, despite the presence of haemolysin-encoding genes in both strains. In line with this, N305 displayed a stronger cytotoxic potential than RF122, as shown by an in vitro MTT assay of MAC-T cells. However, RF122 has been described as a strain that produces high levels of α-haemolysin [55]. This discrepancy between the genomic and proteomic profiles and the expected cytotoxicity phenotype underlines the importance of the in vivo validation of in silico and proteomic analyses. The virulence of *S. aureus* is obviously associated with its gene content, but also with its capacity to express this genetic equipment in a specific context. The differences observed here therefore advocated for more severe mastitis with RF122.

**Table 3:** **Compilation of potentially virulence associated proteins identified in this work**

		**Access.** ^**c**^				**Method** ^**f**^		
**Name of the protein** ^**a**^	**Locus name** ^**b**^	**RF122**	**N305**	**Mass** ^**d**^	**Loc.** ^**e**^	**Exo.**	**Tot.**	**Shav.**
**CELLULAR PROCESSES AND SIGNALING**								
**Cell cycle control, cell division, chromosome partitioning**								
Probable transglycosylase IsaA	*isaA*	Q2YWD9	J0L078	28.2	S	RF122		RF122, N305
**Cell wall/membrane/envelope biogenesis**								
Iron-regulated surface determinant protein A	*isdA*	Q2YX95	J0KNZ4		W			RF122, N305
Iron-regulated surface determinant protein B	*isdB*	Q2YX96	J1ET39	72.0	W	RF122		RF122, N305
Iron-regulated surface determinant protein C	*isdC*	Q2YX94	J0KVY4					RF122, N305
Iron-regulated protein	*isdD*	Q2YX93	J0UGM1	41.2	U			RF122
Lipoteichoic acid synthase	*ltaS*	Q2YSL2	J1EW90	74.2	C/M	RF122		
Penicillin-binding protein 2	*pbp2*	Q2YY56	J1EUU3	80.2	C/M			RF122
**Post-translational modification, protein turnover, and chaperones**								
Serine protease SplE	*splE*	Q2YTM5	J1EZ78	25.6	C/M	N305		
Serine protease SplF	*splF*	Q2YTM6	J0KV93	25.6	S	N305		
Serine protease SplD	*splD*		J0L226	25.6	S	N305		
Serine proteinase (SplB)	Newbould 305_0049	Q2YTM3	J0UNB6	27.8	S	N305		
Chaperone protein DnaK	*dnaK*	Q2YT47	J1EZS6	66.2	C		RF122	RF122, N305
Alkyl hydroperoxide reductase subunit F	*ahpF*	Q2YVH8	J0KUQ5	54.5	C/M		RF122	RF122, N305
Alkyl hydroperoxide reductase subunit C	*ahpC*	Q2YVK2	J1EYK8	20.9	C		RF122	RF122, N305
Trigger factor	*tig*	Q2YTB4	J0KVL9	48.4	C		N305	RF122, N305
**Defense mechanisms**								
Alkaline shock protein 23	*asp23*	Q2YYG3	J0KY19	18.6	U		N305	RF122, N305
**INFORMATION STORAGE AND PROCESSING**								
**Replication, recombination and repair**								
Thermonuclease	*nucI*	Q2YXU2	J0UHM2	21.8	S			RF122, N305
**Translation, ribosomal structure and biogenesis**								
Elongation factor Ts	*tsf*	Q2YXL1	J0KWZ6	32.3	C	N305	RF122	N305
Elongation factor Tu	*tuf*	Q2YSB3	J1EVM7	43.0	C		N305	RF122, N305
**METABOLISM**								
**Carbohydrate transport and metabolism**								
Glyceraldehyde-3-phosphate dehydrogenase	*gap*	Q2YSF2	J0KSH5	36.2	C	N305		RF122, N305
Glucose-6-phosphate isomerase	*pgi*	Q2YWS3	J0UKP5	49.7	C	N305		
Enolase	*eno*	Q2YSE8	J1EWJ2	47.0	C	N305		RF122, N305
Phosphoglycerate kinase	*pgk*	Q2YSF1	J1EWC9	42.5	C	N305	N305	RF122, N305
**Inorganic ion transport and metabolism**								
Superoxide dismutase M	*sodM*	Q2YUU9	J0UM22	22.9	S	N305		
**POORLY CHARACTERIZED**								
Gamma-hemolysin component A	*hlgA*	Q2YVZ2	J0KNX1	36.3	S			N305
Gamma-hemolysin component B	*hlgB*	Q2YVZ0	J0KVW4	36.6	S			RF122, N305
Gamma-hemolysin component C	*hlgC*	Q2YVZ1	J1ET20	35.5	S	N305		
Staphylococcal enterotoxin C-bovine	*sec-bov*	Q2YVN9		31.2	S	RF122		
Superantigen-like protein 7	*set5*	Q2YVR9	J1EYV6	26.0	S	N305		
Superantigen-like protein	*set11*	Q2YVM7	J1EYV1	25.7	S	N305		
Superantigen-like protein	*set19*		J0UMW3	39.8	S			N305
Superantigen-like protein	Newbould 305_1808	Q2YXF4	J0KPN6	27.7	S			N305
Leukocidin F subunit	*lukA*	Q2YU84	J1ET76	38.6	S			RF122, N305
Leukocidin S subunit	*lukB*	Q2YU83	J0KP92	40.2	S			N305
Panton-Valentine leukocidin LukF’-PV chain	*lukF’-PV*	Q2YWM7		36.4	S			RF122
Leukocidin chain lukM	*lukM*	Q2YWM8		35.0	S			RF122
Leukotoxin E subunit	*lukE*	Q2YTQ3	J0L217	34.6	S			RF122, N305
Extracellular matrix and plasma binding protein	Newbould 305_1324		J0UKH8	38.3	W			N305
Uncharacterized protein (*vwb* ^*Sbo5*^-like)	Newbould 305_0962		J0KUT2	57.4	S			N305
Immunoglobulin G binding protein A (Protein A)	*spa*		J1EXZ6	56.8	W			N305
Elastin-binding protein EbpS	*ebpS*	Q2YY76	J0KQY7	52.9	C/M			RF122, N305
Molecular chaperone Hsp31 and glyoxalase 3	*hchA*		J1EVT7	32.0	C		N305	

^a^Proteins are classified in COG. Names are given according to annotation of genome sequences

^b^Correspond to the commonly found name of the gene.

^c^Accessions numbers are given according to references on UniProtKB [35].

^d^ Theoretical mass (in kDa) as predicted from the protein sequence [36].

^e^Predicted localization based on PSORTb software. S = Extracellular C = Cytoplasmic C/M = Cytoplasmic/Membrane W = Cell wall U = Unknown.

^f^Method that enabled the observation of the protein with the name of the strain in which the protein was identified: Exo. = Proteins identified in supernatant Tot. =Proteins identified in total lysate Shav. =Proteins present in trypsin shaving solution.

Interestingly, SaPI-N305_3, one of the SaPIs predicted in silico by PIPS analysis, was absent from the RF122 genome and shared homology with the previously described SaPIbov4 and SaPIbov5 [56]. This putative SaPI carries the Newbould 305_0962 gene whose product was found to be surface-exposed by trypsin shaving in N305. This gene shares homology with SaPIbov4_ORF15, a gene encoding a bovine and SaPI-associated variant of von Willebrand factor binding protein which has been described in the *Staphylococcus aureus* strain BA4 pathogenicity island SaPIbov4 [57]. These bovine variants of vWbp (identified in SaPIbov4 and SaPIbov5) have been shown to provide carrier *S. aureus* strains with an ability to specifically coagulate ruminant plasma, whereas strains that do not carry these SaPIs (such as RF122) coagulate rabbit but not ruminant plasma [56]. We were able to show that N305 did indeed clot ruminant (bovine and caprine) plasma. This capacity to coagulate plasma is correlated with an ability to induce abscesses during infections [58]. The formation of abscesses is a mechanism of bacterial resistance against host immune cells. N305 is therefore endowed with ruminant-specific coagulation capacities which reflect its adaptation to the bovine host and correlate with its propensity to induce chronic mastitis.

### *S. aureus* N305 is suitably armed to invade host cells

A genomic comparison of *S. aureus* N305 and RF122 also revealed differences in colonization and invasion factors. Most of these factors belong to surface proteins such as Secretable Expanded Repertoire Adhesive Molecules (SERAMs) or MSCRAMMs, which participate in both adhesion and the internalization of *S. aureus* in host cells [59]. SERAMs or MSCRAMMs interact with host proteins such as fibronectin, collagen and elastin, and trigger invasion. Numerous studies have reported the importance of these factors during an infection. In particular, fibronectin-binding proteins are considered to be the principal staphylococcal proteins involved in *S. aureus* internalization in host cells [60]. The analysis of the N305 genome using the SurfG + package [27] enabled the identification of PSE proteins. When the genome comparison of RF122 and N305 was focused on these PSE proteins, it revealed that N305 is better equipped than RF122 to achieve host invasion. *S. aureus* N305 indeed possesses two fibronectin-binding proteins (*fnbA* and *fnbB*) rather than one for RF122 (*fnbA*). Furthermore, a comparison of the predicted FnbA protein sequence revealed that N305 FnbA presented two additional fibronectin-binding domains, and RF122 FnbA only one. This suggests a greater affinity of N305 for fibronectin. It should be noted that fibronectin-binding proteins have been shown to be indispensable to the internalization of *S. aureus* into host cells [60,61]. Other surface protein genes were found in N305 while they were missing from the RF122 genome.

Biofilm formation was not significantly different in the two strains, in the conditions used (on abiotic surface). Although *bap* gene is reportedly associated with chronic mastitis isolates [8], its absence does not hinder N305 to induce chronic mastitis. Moreover, the *sasG* and *pls* genes, known to participate in host colonization and modulate pathogen internalization, were also found in N305 only. These observations correlate well with previous studies by our laboratory which compared the adhesion and internalization capacities of both strains, in vitro, on bovine MEC cultures (MAC-T line). The adhesion and internalization rates of N305 were indeed significantly higher than those of RF122 [24]. These capacities seem to favour host invasion by *S. aureus* N305, and together with the smaller number of toxin genes and host-adaptation features such as production of the bovine variant of vWbp, may account for a milder but chronic phenotype in N305 mastitis.

Some moonlighting proteins were also found in the proteomic profiles. Proteins identified as being relatively more abundant in the N305 secretome formed part of the “Carbohydrate transport and metabolism” COG category and were also involved in adhesion and colonization of host tissue, as has been reported for enolase [62,63], glyceraldehyde-3-phosphate deshydrogenase (GAPDH) [64] and phosphoglycerate kinase (*pgk*) [65]. Other cytoplasmic proteins reportedly involved in adhesion, such as glucose-6-phosphate isomerase (*pgi*) [66], fructose-biphosphate aldolase (*fda*) [67], superoxide dismutase (*sod*) [68] and elongation factor Tu (*tuf*) [69], were also found, but in the N305 supernatant only (Figure 4). The fact that N305 expresses more of these proteins than RF122 may also account for N305′s better capacities to adhere, internalize and cause chronic infections.

### Conclusions

N305 harbours fewer toxin genes but more colonization and invasion factors than RF122. In vitro, coagulation, proteolysis and cytotoxicity were significantly higher in N305 compared to RF122 as well as adhesion and internalisation, as previously demonstrated in Bouchard et al. [24]. This correlated well with its gene content and proteomic profile. The range of bovine-adaptive features might account for the propensity of N305 to persistence and mild and chronic mastitis, which is more difficult to detect and cure. By contrast, RF122 appeared to be less well equipped in terms of its genes related to host-adaptation and tissue invasion, but better equipped in toxin genes, and therefore more prone to inducing a severe inflammatory response. Whether these two profiles correspond to the evolutionary strategies of *S. aureus* bovine strains towards a commensal or strictly pathogenic lifestyle is not yet known, and this point deserves further investigation, including epidemiological studies. In line with this, based on MLST data, Smith et al. suggested that N305 should be considered as a teat skin associated strains [70]. *S. aureus* N305 nevertheless represents a strain of choice for further study to improve our understanding of the pathogenesis of *S. aureus* in the context of chronic mastitis. A complete demonstration of the involvement of the determinants identified here would require further experiments such as gene disruption and virulence determination in vivo. The genome sequence and fine characterization of this strain is a first necessary step towards developing strategies to understand, prevent and combat this disease.

## Additional files

Additional file 1:
**CDS classifications of** ***S. aureus*** **Newbould 305 and RF122 in the biological process.** The assignment of protein functions to the ORFs of the N305 (A) and RF122 (B) genomes was performed manually using the results from BLASTP and the COG (Clusters of Orthologous Groups). The percentage of genes encoding transposases and their inactivated derivatives (COG L) was 5.5% of the total genes with COGs classifications in Newbould 305. Additional file 2:
**Proteins identified with the trypsin shaving method.** This table presents the proteins identified by mass spectrometry after a trypsin treatment of bacterial surface (see Materials and methods for details) for RF122 and N305 strains. Additional file 3:
**Proteolytic activity of Newbould 305 and RF122 supernatants assessed on PCA agar medium supplemented with 5% skimmed milk.** Proteolysis assays on PCA gelose added with 5% of skimmed milk. Culture supernatants of *S. aureus* RF122 (A) and Newbould 305 (B) were concentrated 10x and filtered before depositing 50 μL in a well. A halo of proteolysis can be observed with N305. Additional file 4:
**Proteins identified as differentially abundant in secretome of** ***S. aureus*** **RF122 and N305.** This table presents the proteins identified by mass spectrometry (see Materials and methods for details) in the culture supernatants of RF122 and N305 strains. Additional file 5:
**Proteins identified as differentially expressed in the total lysate by** ***S. aureus*** **RF122 and N305.** This table presents the proteins identified by mass spectrometry (see Materials and methods for details) in the whole cell lysates of RF122 and N305 strains.
