# Supplementary figures and images for: Fine-tuned characterization of Staphylococcus aureus Newbould 305, a strain associated with mild and chronic mastitis in bovines
Source: Vet Res. 2014 Oct 14;45(1):106. doi: 10.1186/s13567-014-0106-7 (PMC4230361; doi:10.1186/s13567-014-0106-7)

## Slide 1
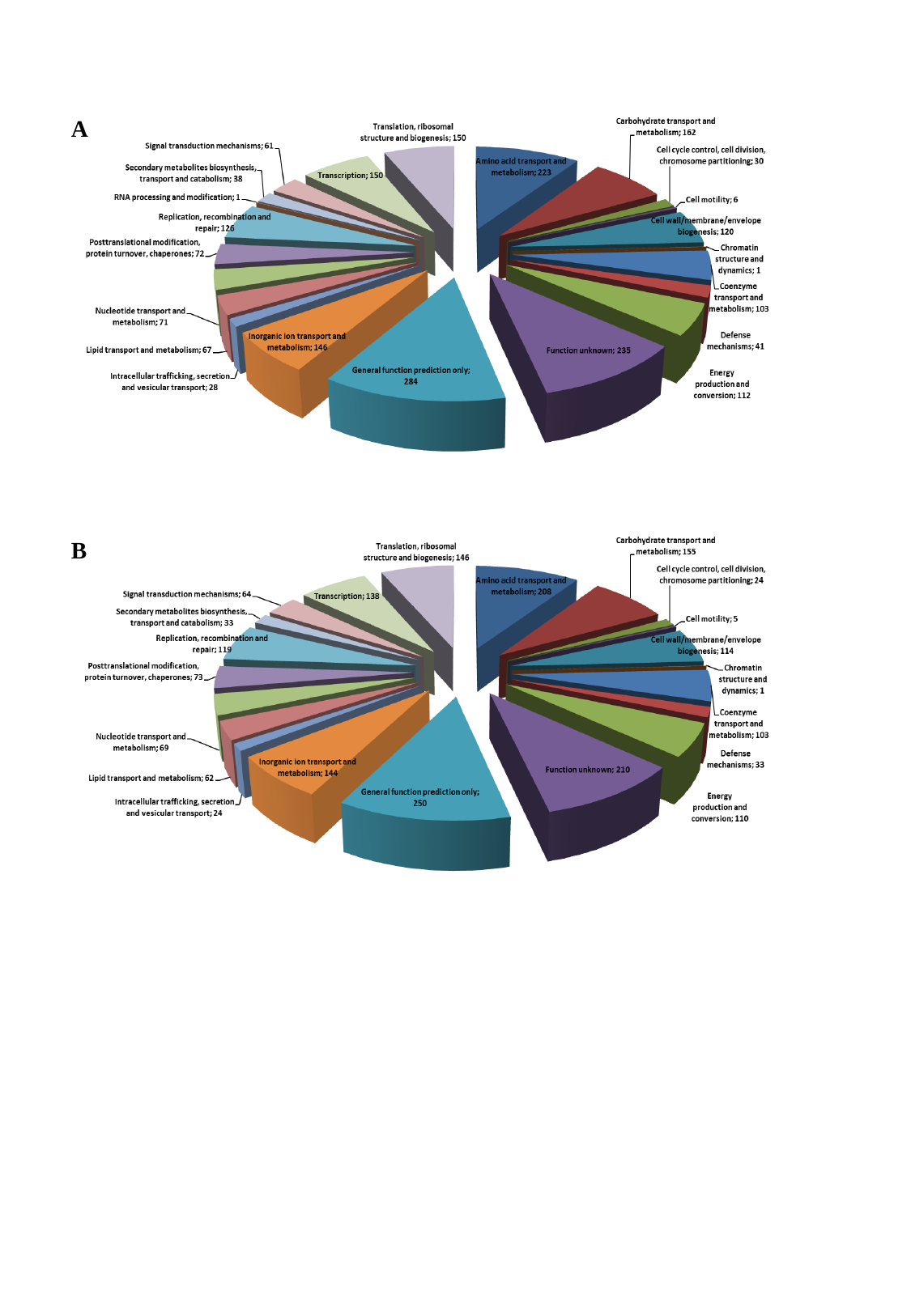

A
B

Supplement: Additional file 1: — CDS classifications of S. aureus Newbould 305 and RF122 in the biological process. The assignment of protein functions to the ORFs of the N305 (A) and RF122 (B) genomes was performed manually using the results from BLASTP and the COG (Clusters of Orthologous Groups). The percentage of genes encoding transposases and their inactivated derivatives (COG L) was 5.5% of the total genes with COGs classifications in Newbould 305. [file 13567_2014_106_MOESM1_ESM.pptx]

## Slide 1
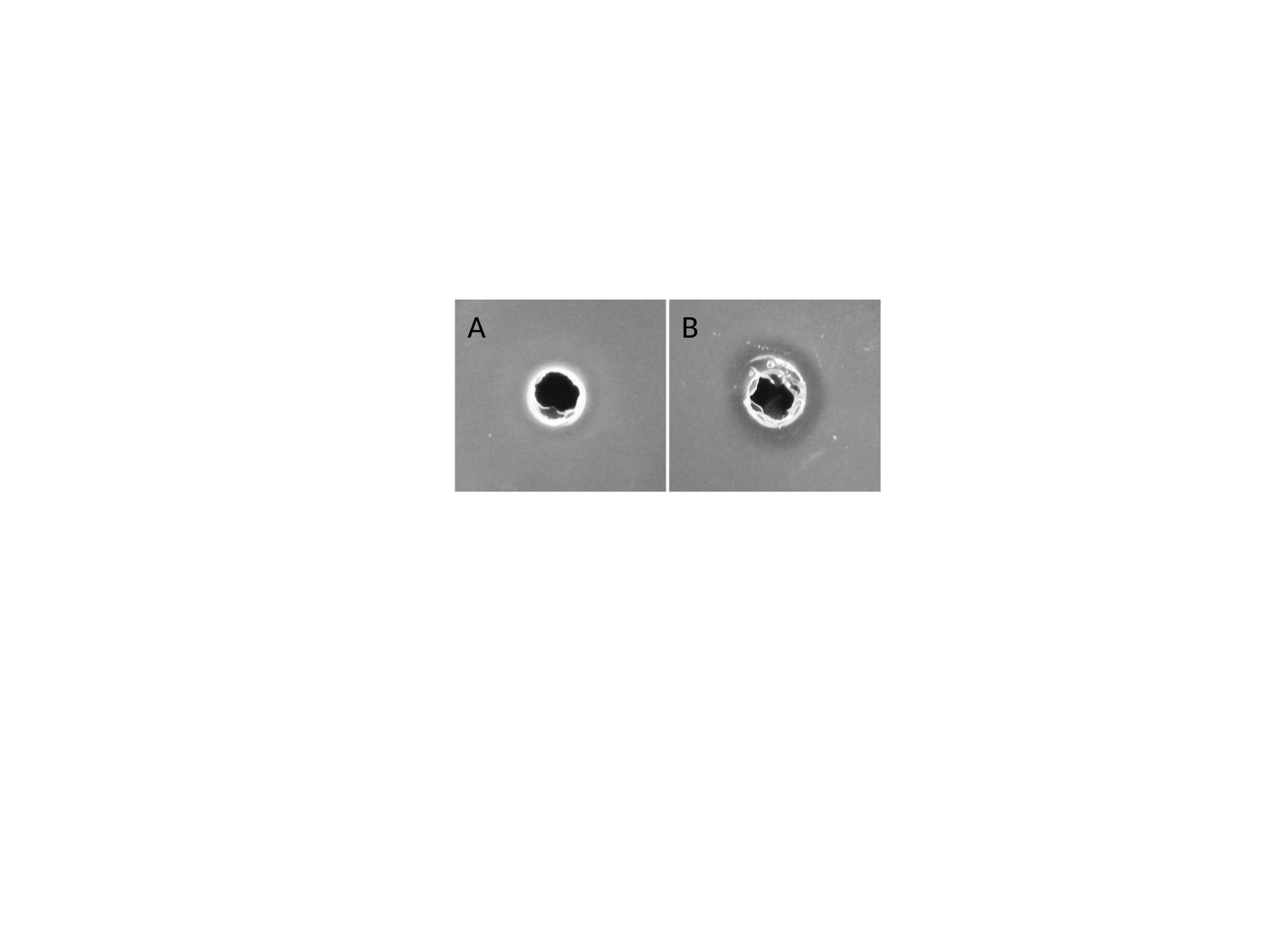

A
B

Supplement: Additional file 3: — Proteolytic activity of Newbould 305 and RF122 supernatants assessed on PCA agar medium supplemented with 5% skimmed milk. Proteolysis assays on PCA gelose added with 5% of skimmed milk. Culture supernatants of S. aureus RF122 (A) and Newbould 305 (B) were concentrated 10x and filtered before depositing 50 μL in a well. A halo of proteolysis can be observed with N305. [file 13567_2014_106_MOESM3_ESM.pptx]
