# Supplementary material for: Fine-tuned characterization of Staphylococcus aureus Newbould 305, a strain associated with mild and chronic mastitis in bovines
Source: Vet Res. 2014 Oct 14;45(1):106. doi: 10.1186/s13567-014-0106-7 (PMC4230361; doi:10.1186/s13567-014-0106-7)
Supplement: Additional file 2: — Proteins identified with the trypsin shaving method. This table presents the proteins identified by mass spectrometry after a trypsin treatment of bacterial surface (see Materials and methods for details) for RF122 and N305 strains. [file 13567_2014_106_MOESM2_ESM.docx]

**Additional file 4 Proteins identified with the trypsin shaving method.**

| Name of proteins identified in RF122 ^a^ | Locus name ^b^ | Access. ^c^ | log(E value) ^d^ | Cover. ^e^ | Mass ^f^ | NP ^g^ | PAI ^h^ | Loc. ^i^ |
| --- | --- | --- | --- | --- | --- | --- | --- | --- |
| **CELLULAR PROCESSES AND SIGNALING** | | | | | | | | |
| **Cell cycle control, cell division, chromosome partitioning** | | | | | | | | |
| Probable transglycosylase IsaA | isaA | Q2YWD9 | -64.45 | 47 | 24.2 | 10 | 4.25 | S |
| **Cell wall/membrane/envelope biogenesis** | | | | | | | | |
| Probable exported protein | SAB2519 | Q2YZ57 | -23.92 | 10 | 69.2 | 4 | 0.29 | S |
| Penicillin-binding protein 2 | pbp2 | Q2YY56 | -19.82 | 14 | 80.2 | 6 | 0.21 | C/M |
| N-acetylmuramoyl-L-alanine amidase sle1 | sle1 | Q2YVT4 | -14.69 | 16 | 35.8 | 4 | 0.38 | W |
| **Post-translational modification, protein turnover, and chaperones** | | | | | | | | |
| Probable thioredoxin | SAB0762c | Q2YWM3 | -5.84 | 24 | 12.0 | 2 | 0.40 | C |
| Probable serine protease | SAB1586 | Q2YTG2 | -14.24 | 16 | 45.6 | 4 | 0.24 | C/M |
| **Signal transduction mechanisms** | | | | | | | | |
| S-ribosylhomocysteine lyase | luxS | Q2YUL9 | -5.47 | 17 | 17.5 | 2 | 0.60 | C |
| Serine-protein kinase RsbW | rsbW | Q2YUI8 | -5.87 | 13 | 17.8 | 2 | 0.20 | C |
| Histidine protein kinase SaeS | saeS | Q2YSM6 | -6.86 | 9 | 39.6 | 2 | 0.25 | C/M |
| **Intracellular trafficking, secretion, and vesicular transport** | | | | | | | | |
| Type-I signal peptidase | spsB | Q2YWR9 | -18.30 | 28 | 21.6 | 5 | 0.46 | W |
| **Defense mechanisms** | | | | | | | | |
| Lipid II:glycine glycyltransferase | femX | Q2YYN5 | -20.68 | 8 | 48.4 | 3 | 0.19 | C |
| **INFORMATION STORAGE AND PROCESSING** | | | | | | | | |
| **Translation, ribosomal structure and biogenesis** | | | | | | | | |
| 30S ribosomal protein S6 | rpsF | Q2YVJ2 | -10.10 | 52 | 11.5 | 5 | 0.86 | C |
| 50S ribosomal protein L28 | rpmB | Q2YXJ2 | -4.47 | 23 | 6.9 | 2 | 1.00 | C |
| 50S ribosomal protein L18 | rplR | Q2YYL3 | -11.99 | 33 | 13.0 | 4 | 1.00 | C |
| 30S ribosomal protein S18 | rpsR | Q2YVJ0 | -15.52 | 32 | 9.2 | 4 | 2.00 | C |
| 50S ribosomal protein L17 | rplQ | Q2YYM3 | -15.45 | 22 | 13.7 | 2 | 0.75 | C |
| 50S ribosomal protein L30 | rpmD | Q2YYL5 | -11.70 | 36 | 6.5 | 2 | 0.50 | C |
| Translation initiation factor IF-3 | infC | Q2YTA9 | -5.42 | 10 | 20.1 | 2 | 0.29 | C |
| Phenylalanine--tRNA ligase alpha subunit | pheS | Q2YX87 | -11.97 | 8 | 40.0 | 2 | 0.17 | C |
| **Transcription** | | | | | | | | |
| Probable exported protein | SAB0922c | Q2YX19 | -18.61 | 26 | 46.0 | 5 | 0.38 | C/M |
| Putative uncharacterized protein | SAB0063 | Q2YUV7 | -30.92 | 29 | 29.5 | 6 | 0.41 | C |
| Transcription termination/antitermination protein nusG | nusG | Q2YSC5 | -6.97 | 13 | 20.6 | 2 | 0.18 | C |
| **Replication, recombination and repair** | | | | | | | | |
| **Additional file 4 (*continued*)** |  |  |  |  |  |  |  |  |
| Name of proteins identified in RF122 ^a^ | Locus name ^b^ | Access. ^c^ | log(E value) ^d^ | Cover. ^e^ | Mass ^f^ | NP ^g^ | PAI ^h^ | Loc. ^i^ |
| DNA ligase | ligA | Q2YU70 | -4.23 | 4 | 74.9 | 2 | 0.05 | C |
| **METABOLISM** | | | | | | | | |
| **Energy production and conversion** | | | | | | | | |
| Probable quinol oxidase subunit 2 | qoxA | Q2YX14 | -20.62 | 20 | 41.7 | 6 | 0.58 | C/M |
| Glycerol-3-phosphate dehydrogenase [NAD(P)+] | gpsA | Q2YY82 | -8.60 | 10 | 36.0 | 3 | 0.25 | C |
| Probable alcohol dehydrogenase | SAB1296c | Q2YY27 | -21.82 | 17 | 35.5 | 4 | 0.54 | U |
| Isocitrate dehydrogenase [NADP] | citC | Q2YTE6 | -7.09 | 6 | 46.3 | 2 | 0.13 | C |
| **Nucleotide transport and metabolism** | | | | | | | | |
| Deoxyribose-phosphate aldolase 1 | deoC1 | Q2YUU4 | -9.97 | 12 | 23.4 | 2 | 0.18 | C |
| Orotate phosphoribosyltransferase | pyrE | Q2YXG3 | -8.59 | 21 | 22.0 | 2 | 0.25 | C |
| **Carbohydrate transport and metabolism** | | | | | | | | |
| Probable uridylyltransferase SAB2052c | SAB2052c | Q2YYH4 | -8.90 | 7 | 44.8 | 2 | 0.10 | C |
| 2.3-bisphosphoglycerate-independent phosphoglycerate mutase | Pgm | Q2YSE9 | -9.22 | 4 | 56.3 | 2 | 0.19 | C |
| Probable exported protein | SAB1633c | Q2YTL2 | -6.55 | 14 | 32.4 | 2 | 0.21 | S |
| **Coenzyme transport and metabolism** | | | | | | | | |
| Demethylmenaquinone methyltransferase | ubiE | Q2YY85 | -10.47 | 10 | 27.2 | 2 | 0.17 | U |
| **Lipid transport and metabolism** | | | | | | | | |
| 1-phosphatidylinositol phosphodiesterase | Plc | Q2YUT1 | -27.62 | 24 | 36.9 | 6 | 0.89 | S |
| **Inorganic ion transport and metabolism** | | | | | | | | |
| Probable membrane protein | SAB1406 | Q2YSZ5 | -7.96 | 15 | 14.7 | 2 | 0.40 | U |
| **POORLY CHARACTERIZED** | | | | | | | | |
| Ser-Asp rich fibrinogen-binding/bone sialoprotein-binding protein | SAB0513 | Q2YSA1 | -232.64 | 45 | 120.7 | 33 | 1.06 | W |
| Panton-Valentine leukocidin LukF-PV chain | SAB0783 | Q2YWM7 | -34.02 | 36 | 36.4 | 8 | 0.77 | S |
| Leukocidin chain lukM | SAB0782 | Q2YWM8 | -35.33 | 29 | 35.0 | 7 | 0.59 | S |
| Putative uncharacterized protein | SAB1759 | Q2YTX0 | -23.39 | 29 | 19.2 | 4 | 1.20 | U |
| Probable lipoprotein | SAB0692 | Q2YSI9 | -12.07 | 14 | 34.3 | 4 | 0.25 | U |
| Iron-regulated protein | isdD | Q2YX93 | -8.81 | 10 | 41.2 | 3 | 0.20 | U |
| Probable lipoprotein | SAB1403 | Q2YSZ8 | -6.23 | 15 | 21.3 | 2 | 0.25 | S |
| Leukotoxin E subunit | lukE | Q2YTQ3 | -20.97 | 20 | 34.6 | 5 | 0.33 | S |
| Elastin-binding protein EbpS | ebpS | Q2YY76 | -27.20 | 10 | 52.9 | 5 | 0.40 | C/M |
| UPF0342 protein SAB1778c | SAB1778c | Q2YTZ2 | -27.39 | 53 | 13.2 | 3 | 0.63 | C |
| Putative uncharacterized protein | SAB0328c | Q2YVI0 | -11.50 | 14 | 23.6 | 3 | 0.33 | U |
| Putative uncharacterized protein | SAB1052 | Q2YXD4 | -14.83 | 20 | 25.5 | 3 | 0.44 | C |
| **Additional file 4 (*continued*)** |  |  |  |  |  |  |  |  |
| Name of proteins identified in N305 ^a^ | Locus name ^b^ | Access. ^c^ | log(E value) ^d^ | Cover. ^e^ | Mass ^f^ | NP ^g^ | PAI ^h^ | Loc. ^i^ |
| **CELLULAR PROCESSES AND SIGNALING** | | | | | | | | |
| **Cell cycle control, cell division, chromosome partitioning** | | | | | | | | |
| Septation ring formation regulator EzrA | ezrA | J1EZG0 | -50.38 | 18 | 66.1 | 6 | 0.24 | C/W |
| Iron-sulfur cluster repair protein ScdA | scdA | J0UMD9 | -8.72 | 17 | 25.4 | 3 | 0.25 | C |
| **Cell wall/membrane/envelope biogenesis** | | | | | | | | |
| Elongation factor 4 | lepA | J1EZS2 | -9.48 | 4 | 68.0 | 2 | 0.08 | C/W |
| Undecaprenyl-PP-MurNAc-pentapeptide-UDPGlcNAc GlcNAc transferase | murG | J1EUR0 | -11.35 | 8 | 39.6 | 2 | 0.13 | C/W |
| UDP-N-acetylenolpyruvoylglucosamine reductase | murB | J0KZ95 | -6.26 | 8 | 33.7 | 2 | 0.15 | C |
| UDP-N-acetylmuramoylalanine--D-glutamate ligase | murD | J0KWM8 | -8.80 | 8 | 49.7 | 2 | 0.18 | C |
| **Post-translational modification, protein turnover, and chaperones** | | | | | | | | |
| ATP-dependent protease subunit HslV | hslV | J0KWZ1 | -20.34 | 19 | 19.5 | 3 | 0.63 | C |
| Thioredoxin reductase | trxB | J1EWC4 | -10.49 | 10 | 33.5 | 2 | 0.20 | C |
| Peptide methionine sulfoxide reductase MsrA | msrA | J0KQP0 | -19.91 | 24 | 20.5 | 3 | 0.50 | U |
| **Signal transduction mechanisms** | | | | | | | | |
| Universal stress protein family protein | Newbould305_0182 | J1EZI3 | -18.69 | 28 | 15.2 | 3 | 0.50 | U |
| Two-component response regulator | yycF | J0L0Q9 | -14.60 | 25 | 27.4 | 4 | 0.42 | C |
| **Intracellular trafficking, secretion, and vesicular transport** | | | | | | | | |
| Signal recognition particle protein | Ffh | J0KPU1 | -20.71 | 10 | 50.6 | 3 | 0.20 | C/W |
| **Defense mechanisms** | | | | | | | | |
| ABC transporter | Newbould305_0374 | J1EWZ5 | -5.71 | 9 | 25.7 | 2 | 0.17 | C/W |
| ATP-binding ABC transporter protein | Newbould305_1348 | J1EWF8 | -9.26 | 8 | 38.1 | 2 | 0.15 | C/W |
| **INFORMATION STORAGE AND PROCESSING** | | | | | | | | |
| **Translation, ribosomal structure and biogenesis** | | | | | | | | |
| Pseudouridine synthase | rluB | J1EUZ6 | -17.69 | 20 | 27.9 | 3 | 0.25 | C |
| Tyrosine--tRNA ligase | tyrS | J1EZG1 | -12.66 | 12 | 47.5 | 3 | 0.19 | C |
| Isoleucine--tRNA ligase | ileS | J0KWN8 | -103.54 | 27 | 104.7 | 18 | 0.59 | C |
| Arginine--tRNA ligase | argS | J0KYW4 | -20.43 | 8 | 62.2 | 3 | 0.10 | C |
| Leucine--tRNA ligase | leuS | J0UNH6 | -18.84 | 10 | 91.6 | 3 | 0.12 | C |
| Peptide chain release factor 3 | prfC | J0KSW0 | -10.23 | 6 | 59.4 | 2 | 0.11 | C |
| Peptide deformylase | Def | J0UGP6 | -6.66 | 12 | 20.5 | 2 | 0.25 | C |
| Uncharacterized protein | Newbould305_0284 | J1EZR2 | -45.86 | 72 | 11.0 | 6 | 2.25 | C |
| UPF0477 protein Newbould305_1457 | Newbould305_1457 | J0KSV1 | -22.60 | 27 | 19.2 | 3 | 0.63 | C |
| **Transcription** | | | | | | | | |
| **Additional file 4 (*continued*)** |  |  |  |  |  |  |  |  |
| Name of proteins identified in N305 ^a^ | Locus name ^b^ | Access. ^c^ | log(E value) ^d^ | Cover. ^e^ | Mass ^f^ | NP ^g^ | PAI ^h^ | Loc. ^i^ |
| Rrf2 family transcriptional regulator | Newbould305_0252 | J1EZQ2 | -34.18 | 44 | 15.5 | 4 | 1.00 | C |
| Redox-sensing transcriptional repressor rex | Rex | J1ET27 | -11.38 | 15 | 23.5 | 3 | 0.30 | C |
| HTH-type transcriptional regulator rot | Rot | J0L278 | -21.52 | 26 | 19.3 | 5 | 1.00 | U |
| Uncharacterized protein | Newbould305_2350 | J0KP60 | -10.22 | 11 | 26.0 | 2 | 0.22 | C |
| **Replication, recombinaison and repair** | | | | | | | | |
| Transcription-repair coupling factor | Newbould305_2621 | J0KVM9 | -6.61 | 2 | 134.0 | 2 | 0.04 | C |
| Thermonuclease | nucI | J0UHM2 | -21.19 | 18 | 21.8 | 4 | 0.63 | S |
| DNA gyrase subunit B | gyrB | J1EXV7 | -12.87 | 9 | 72.4 | 4 | 0.14 | C |
| Chromosomal replication initiator protein DnaA | dnaA | J0KTV1 | -11.80 | 9 | 51.8 | 3 | 0.11 | C |
| Primosomal protein DnaI | dnaI | J0UNP2 | -11.74 | 10 | 35.5 | 2 | 0.13 | C |
| DNA polymerase | polA | J1EZI5 | -7.59 | 3 | 99.0 | 2 | 0.05 | C |
| **METABOLISM** | | | | | | | | |
| **Energy production and conversion** | | | | | | | | |
| Glycerol kinase | glpK | J0KX39 | -16.25 | 10 | 55.5 | 4 | 0.17 | C |
| 2.3-bisphosphoglycerate-independent phosphoglycerate mutase | Pgm | J0KZB7 | -13.30 | 5 | 56.3 | 2 | 0.13 | C |
| Phosphotransacetylase | eutD | J0UJS8 | -6.41 | 13 | 34.8 | 2 | 0.17 | C |
| Dihydrolipoyl dehydrogenase | pdhD | J0UGP2 | -11.47 | 9 | 49.3 | 3 | 0.17 | C |
| Zinc-containing alcohol dehydrogenase superfamily protein | Newbould305_1739 | J0KRK1 | -10.76 | 7 | 36.5 | 2 | 0.17 | C |
| **Amino acid transport and metabolism** | | | | | | | | |
| Probable glycine dehydrogenase [decarboxylating] subunit 2 | gcvPB | J0UP14 | -30.75 | 30 | 54.7 | 6 | 0.43 | C |
| Arginine deiminase | arcA | J0L0F5 | -10.33 | 11 | 46.8 | 4 | 0.16 | C |
| Probable glycine dehydrogenase [decarboxylating] subunit 1 | gcvPA | J1EZX4 | -16.83 | 14 | 49.6 | 3 | 0.25 | C |
| Proline dipeptidase | Newbould305_2310 | J0UJ09 | -22.99 | 14 | 39.2 | 4 | 0.44 | C |
| Bifunctional 3-deoxy-7-phosphoheptulonate synthase/chorismate mutase | aroA | J1EZF3 | -18.68 | 23 | 40.5 | 4 | 0.45 | C |
| Aminopeptidase | Newbould305_2446 | J1ETE6 | -8.35 | 8 | 46.8 | 3 | 0.14 | C |
| Cytosol aminopeptidase | Newbould305_1382 | J0KSP0 | -10.49 | 9 | 54.0 | 3 | 0.15 | C |
| Histidinol-phosphate aminotransferase | hisC | J1EW94 | -9.73 | 7 | 39.7 | 2 | 0.14 | U |
| Uncharacterized protein | Newbould305_0930 | J0L1M4 | -20.58 | 26 | 21.2 | 4 | 0.40 | U |
| **Nucleotide transport and metabolism** | | | | | | | | |
| Phosphoribosylamine--glycine ligase | purD | J1EX21 | -59.75 | 30 | 45.7 | 9 | 0.56 | C |
| Deoxyribose-phosphate aldolase | deoC1 | J0UM27 | -10.78 | 12 | 23.4 | 2 | 0.18 | C |
| Bifunctional protein PyrR | pyrR | J1ETU6 | -25.30 | 40 | 19.0 | 6 | 0.50 | C |
| Phosphoribosylaminoimidazole carboxylase ATPase subunit | purK | J0KSZ4 | -11.19 | 10 | 42.3 | 3 | 0.19 | C/W |
| **Additional file 4 (*continued*)** |  |  |  |  |  |  |  |  |
| Name of proteins identified in N305 ^a^ | Locus name ^b^ | Access. ^c^ | log(E value) ^d^ | Cover. ^e^ | Mass ^f^ | NP ^g^ | PAI ^h^ | Loc. ^i^ |
| Phosphoribosylformylglycinamidine synthase, purS | Newbould305_1509 | J0UKY0 | -8.85 | 27 | 9.8 | 2 | 0.40 | C |
| **Carbohydrate transport and metabolism** | | | | | | | | |
| Aerobic glycerol-3-phosphate dehydrogenase | glpD | J1EU92 | -19.57 | 7 | 62.2 | 4 | 0.18 | C |
| Phosphoglucomutase | Newbould305_2517 | J1ESV1 | -8.71 | 4 | 61.5 | 2 | 0.07 | C |
| **Lipid transport and metabolism** | | | | | | | | |
| Isoprenyl transferase | uppS | J1ETW9 | -6.18 | 18 | 29.8 | 2 | 0.27 | C |
| 2-C-methyl-D-erythritol 4-phosphate cytidylyltransferase | ispD | J0L191 | -18.25 | 20 | 26.6 | 4 | 0.53 | U |
| Cardiolipin synthase | Cls | J1EU91 | -6.68 | 4 | 56.3 | 2 | 0.11 | C/W |
| Carboxylesterase | Newbould305_2553 | J0KNT7 | -11.33 | 7 | 51.9 | 3 | 0.18 | C |
| FMN-dependent NADH-azoreductase | azoR | J1EY78 | -22.41 | 23 | 23.3 | 4 | 0.33 | C |
| **Coenzyme transport and metabolism** | | | | | | | | |
| Lipoate-protein ligase A | Newbould305_1469 | J0UKU6 | -25.40 | 38 | 37.8 | 5 | 0.75 | C |
| Glutamine amidotransferase subunit PdxT | pdxT | J1EVJ4 | -10.82 | 24 | 20.5 | 3 | 0.27 | C |
| Phosphomethylpyrimidine kinase | Newbould305_1105 | J0KYU1 | -9.42 | 8 | 29.8 | 2 | 0.15 | U |
| 2-amino-3-ketobutyrate coenzyme A ligase | Kbl | J0KYQ9 | -5.93 | 6 | 42.8 | 2 | 0.10 | C |
| 3-methyl-2-oxobutanoate hydroxymethyltransferase | panB | J0L0A9 | -5.58 | 9 | 28.1 | 2 | 0.18 | U |
| **Inorganic ion transport and metabolism** | | | | | | | | |
| Toxic ion resistance protein | Newbould305_2180 | J0UHQ3 | -17.75 | 8 | 43.3 | 2 | 0.08 | U |
| Potassium uptake protein | Newbould305_1530 | J0KZY5 | -19.37 | 27 | 24.2 | 3 | 0.71 | C |
| Sulfite reductase flavoprotein | cysJ | J0L0D8 | -6.24 | 4 | 71.5 | 2 | 0.10 | C/W |
| Uncharacterized protein | Newbould305_1190 | J0KZ16 | -17.16 | 13 | 23.6 | 2 | 0.25 | C |
| Iron compound ABC transporter, iron compound-binding protein | Newbould305_2377 | J0UGV2 | -22.82 | 17 | 34.7 | 4 | 0.27 | C/W |
| Aerobactin biosynthesis protein | Newbould305_1550 | J0UJ25 | -34.20 | 16 | 75.9 | 8 | 0.28 | C |
| Cysteine synthase A protein | Newbould305_0655 | J1EY01 | -20.51 | 21 | 35.8 | 4 | 0.45 | C |
| **Secondary metabolites biosynthesis, transport, and catabolism** | | | | | | | | |
| Surfactin/siderophore synthetase | Newbould305_0718 | J0UM69 | -6.84 | 1 | 273.8 | 3 | 0.04 | C |
| Surface protein | Newbould305_0674 | J0L0X0 | -61.75 | 52 | 26.4 | 9 | 2.43 | W |
| **POORLY CHARACTERIZED** | | | | | | | | |
| Uncharacterized protein | Newbould305_1141 | J1EW07 | -34.43 | 45 | 18.5 | 6 | 1.17 | U |
| UPF0042 nucleotide-binding protein Newbould305_1300 | Newbould305_1300 | J0KZB2 | -15.86 | 20 | 34.7 | 5 | 0.33 | C |
| Putative ATP-dependent protease protein | clpB | J0KSR9 | -26.17 | 10 | 98.2 | 4 | 0.13 | C |
| Uncharacterized protein | Newbould305_1086 | J1EVU8 | -23.52 | 40 | 20.8 | 4 | 0.40 | U |
| Uncharacterized protein | Newbould305_2029 | J1EU25 | -9.56 | 44 | 8.1 | 2 | 3.00 | U |
| **Additional file 4 (*continued*)** |  |  |  |  |  |  |  |  |
| Name of proteins identified in N305 ^a^ | Locus name ^b^ | Access. ^c^ | log(E value) ^d^ | Cover. ^e^ | Mass ^f^ | NP ^g^ | PAI ^h^ | Loc. ^i^ |
| Uncharacterized N-acetyltransferase Newbould305_1819 | Newbould305_1819 | J1ETJ0 | -11.42 | 34 | 16.9 | 3 | 0.67 | U |
| Uncharacterized protein | Newbould305_0962 | J0KUT2 | -4.97 | 3 | 57.4 | 2 | 0.09 | S |
| Uncharacterized protein | Newbould305_1304 | J0UKG2 | -6.23 | 7 | 34.2 | 2 | 0.13 | U |
| UPF0297 protein Newbould305_0263 | Newbould305_0263 | J0KVR6 | -4.37 | 28 | 10.2 | 2 | 0.50 | U |
| Probable transcriptional regulatory protein | Newbould305_1196 | J1EW64 | -18.29 | 23 | 26.2 | 3 | 0.83 | C |
| Lipoprotein | Newbould305_2546 | J0KVT0 | -9.85 | 17 | 17.2 | 2 | 0.33 | U |
| GTPase obg | obgE | J0UNR6 | -7.85 | 6 | 47.1 | 2 | 0.08 | C |
| Gamma-hemolysin component A | hlgA | J0KNX1 | -9.58 | 17 | 36.3 | 3 | 0.23 | S |
| Leukocidin S subunit | Newbould305_2380 | J0KP92 | -109.17 | 46 | 40.2 | 18 | 1.88 | S |
| Leukocidin F subunit | Newbould305_2381 | J1ET76 | -84.54 | 38 | 38.6 | 11 | 1.27 | S |
| Superantigen-like protein | set19 | J0UMW3 | -9.75 | 20 | 39.8 | 4 | 0.35 | S |
| Extracellular matrix and plasma binding protein | Newbould305_1324 | J0UKH8 | -60.09 | 31 | 38.3 | 13 | 1.45 | W |
| Superantigen-like protein | Newbould305_1808 | J0KPN6 | -21.78 | 18 | 27.7 | 3 | 0.44 | S |
| Ribosome biogenesis GTPase A | rbgA | J0KQ05 | -6.98 | 15 | 33.3 | 2 | 0.20 | C |
| PilT domain-containing protein | Newbould305_1050 | J0KYN4 | -19.80 | 21 | 38.8 | 3 | 0.36 | C/W |
| Immunoglobulin G binding protein A (Protein A) | Spa | J1EXZ6 | -184.02 | 43 | 56.8 | 20 | 2.16 | W |
| Alkaline shock protein 23 | asp23 | J0KY19 | -8.16 | 18 | 19.1 | 2 | 0.29 | U |
| tRNA modification GTPase MnmE | mnmE | J0ULU6 | -9.88 | 5 | 51.2 | 2 | 0.14 | C |
| 7-cyano-7-deazaguanine synthase | queC | J0KSC2 | -10.29 | 11 | 24.8 | 2 | 0.17 | C |
| 5'-nucleotidase, lipoprotein e(P4) family protein | Newbould305_0857 | J1EYC5 | -10.45 | 15 | 33.3 | 3 | 0.57 | U |
| GTP cyclohydrolase FolE2 | folE2 | J0KRT2 | -10.18 | 14 | 33.3 | 3 | 0.19 | C |

^a^ Proteins of each strains are classified in COG. Names are given according to annotation of genome sequences

^b^ Correspond to the commonly found name of the gene

^c^ Accessions numbers are given according to references on UniProtKB [35]

^d^ Probability of critical error in the protein identification given by the X!Tandem software [36]

^e^ % of the protein covered with the identified peptides

^f^ Theoretical mass as predicted from the protein sequence

^g^ Number of identified peptides

^h^ Protein abundance index ([1])

^i^ Predicted localisation based on PSORTb software. S = Extracellular C = Cytoplasmic C/M = Cytoplasmic/Membrane W = Cell wall U = Unknown
