# Supplementary material for: Fine-tuned characterization of Staphylococcus aureus Newbould 305, a strain associated with mild and chronic mastitis in bovines
Source: Vet Res. 2014 Oct 14;45(1):106. doi: 10.1186/s13567-014-0106-7 (PMC4230361; doi:10.1186/s13567-014-0106-7)
Supplement: Additional file 4: — Proteins identified as differentially abundant in secretome of S. aureus RF122 and N305. This table presents the proteins identified by mass spectrometry (see Materials and methods for details) in the culture supernatants of RF122 and N305 strains. [file 13567_2014_106_MOESM4_ESM.docx]

**Additional file 2 Proteins identified as differentially abundant in secretome of *S. aureus* RF122 and N305.**

| Name of proteins identified in RF122 ^a^ | Spot ^b^ | *p*-value ^c^ | Fold ^d^ | Locus ^e^ | Access. ^f^ | log(E value) ^g^ | Cover. ^h^ | Mass ^i^ | NP ^j^ | PAI ^k^ | Loc. ^l^ |
| --- | --- | --- | --- | --- | --- | --- | --- | --- | --- | --- | --- |
| **CELLULAR PROCESSES AND SIGNALING** | | | | | | | | | | | |
| **Cell cycle control, cell division, chromosome partitioning** | | | | | | | | | | | |
| Probable transglycosylase IsaA | 22 | 4.99E-06 | 2.1 | isaA | Q2YWD9 | -19,11 | 26 | 28,2 | 4 | 0.83 | S |
| **Cell wall/membrane/envelope biogenesis** | | | | | | | | | | | |
| Iron-regulated surface determinant protein B | 1, 2, 3, 4, 5, 6 | 3.93E-05 | 2.4 | isdB | Q2YX96 | -110,18 | 39 | 72,0 | 25 | 1.22 | W |
| Lipoteichoic acid synthase | 7 | 4.53E-04 | 2.1 | ltaS | Q2YSL2 | -71,63 | 33 | 74,2 | 16 | 1.05 | C/M |
| N-acetylmuramoyl-L-alanine amidase | 14, 16 | 7.29E-05 | 2.4 | SAB2519 | Q2YZ57 | -45,94 | 21 | 69,2 | 11 | 0.62 | S |
| **METABOLISM** | | | | | | | | | | | |
| **Nucleotide transport and metabolism** | | | | | | | | | | | |
| Inosine-5'-monophosphate dehydrogenase | 19 | 1.05E-05 | 2.2 | guaB | Q2YVL6 | -22,08 | 13 | 52,7 | 5 | 0.20 | C |
| **Inorganic ion transport and metabolism** | | | | | | | | | | | |
| Probable zinc-binding lipoprotein | 8 | 2.79E-04 | 2 | SAB2286c | Q2YW05 | -15,12 | 14 | 59,0 | 6 | 0.35 | C/M |
| **Secondary metabolites biosynthesis, transport, and catabolism** | | | | | | | | | | | |
| D-alanine--poly(phosphoribitol) ligase subunit 1 | 18 | 4.14E-04 | 2.6 | dltA | Q2YWQ8 | -14,13 | 10 | 54,5 | 3 | 0.18 | C |
| **POORLY CHARACTERIZED** | | | | | | | | | | | |
| Glycerol ester hydrolase | 1, 2 | 9.76E-05 | 2.4 | geh | Q2YVD0 | -42,68 | 22 | 72,2 | 9 | 0.42 | S |
| Staphylococcal enterotoxin C-bovine | 9, 10, 11, 12, 13 | 1.08E-06 | 3.8 | sec-bov | Q2YVN9 | -80,55 | 57 | 31,2 | 20 | 2.00 | S |
| Probable exported protein | 17, 18 | 4.14E-04 | 2.6 | SAB0387 | Q2YVR3 | -15,00 | 8 | 56,3 | 4 | 0.19 | U |
|  |  |  |  |  |  |  |  |  |  |  |  |

| **Additional file 2 (*continued*)** | | | | | | | | | | | |
| --- | --- | --- | --- | --- | --- | --- | --- | --- | --- | --- | --- |
| Name of proteins identified in N305 ^a^ | Spot ^b^ | *p*-value ^c^ | Fold ^d^ | Locus ^e^ | Access. ^f^ | log(E value) ^g^ | Cover. ^h^ | Mass ^i^ | NP ^j^ | PAI ^k^ | Loc. ^l^ |
| **CELLULAR PROCESSES AND SIGNALING** | | | | | | | | | | | |
| **Post-translational modification, protein turnover, and chaperones** | | | | | | | | | | | |
| Foldase protein PrsA | 40 | 4.21E-07 | 6.6 | prsA | J0L203 | -56.29 | 34 | 35.5 | 15 | 1.07 | C/M |
| Coenzyme A disulfide reductase | 29 | 2.21E-03 | 2 | cdr | J1EWL3 | -62.67 | 44 | 49.2 | 14 | 0.63 | C |
| Alkyl hydroperoxide reductase subunit C | 32, 34, 35, 36, 51, 52 | 1.28E-04 | 2 | ahpC | J1EYK8 | -19.09 | 27 | 20.9 | 3 | 0.30 | C |
| Serine protease SplE | 47, 48, 49 | 2.57E-04 | 3.3 | splE | J1EZ78 | -10.09 | 10 | 25.6 | 2 | 0.25 | C/M |
| Serine protease SplF | 48, 49 | 2.57E-04 | 3.3 | splF | J0KV93 | -56.13 | 72 | 25.6 | 15 | 1.89 | S |
| Serine protease SplD | 48 | 2.57E-04 | 3.3 | splD | J0L226 | -46.44 | 59 | 25.6 | 12 | 1.78 | S |
| Serine proteinase (SplB) | 47 | 1.07E-04 | 3.2 | Newbould305_0049 | J0UNB6 | -65.13 | 45 | 27.8 | 16 | 2.56 | S |
| **INFORMATION STORAGE AND PROCESSING** | | | | | | | | | | | |
| **Translation, ribosomal structure and biogenesis** | | | | | | | | | | | |
| Elongation factor Ts | 36, 37, 42 | 1.51E-05 | 2 | tsf | J0KWZ6 | -91.77 | 68 | 32.4 | 21 | 1.56 | C |
| **METABOLISM** | | | | | | | | | | | |
| **Energy production and conversion** | | | | | | | | | | | |
| Pyruvate dehydrogenase E1 component alpha subunit | 32 | 7.32E-04 | 2.4 | pdhA | J0KP28 | -8.58 | 7 | 41,3 | 2 | 0.15 | C |
| L-lactate dehydrogenase | 50, 51, 52 | 2.44E-04 | 2.4 | ldh | J1EXA2 | -31.90 | 22 | 34,3 | 6 | 0.54 | C |
| **Amino acid transport and metabolism** | | | | | | | | | | | |
| Xaa-Pro dipeptidase-like protein | 32 | 7.32E-04 | 2.4 | Newbould305_0180 | J0UNM4 | -9.93 | 9 | 39,5 | 3 | 0.30 | C |
| Thimet oligopeptidase | 25 | 1.53E-03 | 2.4 | pepB | J1EWN7 | -46.18 | 23 | 69,7 | 12 | 0.60 | C |
| **Carbohydrate transport and metabolism** | | | | | | | | | | | |
| Glyceraldehyde-3-phosphate dehydrogenase | 32, 34, 35, 36 | 4.15E-06 | 4.1 | gap | J0KSH5 | -17.63 | 22 | 36,2 | 5 | 0.46 | C |
| Glucose-6-phosphate isomerase | 30 | 2.18E-05 | 2.7 | pgi | J0UKP5 | -101.03 | 55 | 49,7 | 22 | 2.06 | C |
| 2,3-bisphosphoglycerate-dependent phosphoglycerate mutase | 43, 44 | 6.54E-05 | 2.8 | gpmA | J1ET24 | -67.89 | 64 | 26,6 | 15 | 1.42 | U |
| UTP--glucose-1-phosphate uridylyltransferase | 32 | 7.32E-04 | 2.4 | Newbould305_1539 | J1EUY4 | -40.81 | 35 | 44,8 | 12 | 0.75 | C |
| Phosphoglycerate kinase | 32, 33 | 7.32E-04 | 2.4 | pgk | J1EWC9 | -86.90 | 55 | 42,5 | 19 | 1.11 | C |
| Enolase | 28, 31 | 3.75E-04 | 2.3 | eno | J1EWJ2 | -104.19 | 57 | 47,0 | 20 | 1.15 | C |
| Fructose-bisphosphate aldolase class 1 | 43 | 6.54E-05 | 2.8 | fda | J1EXA7 | -83.88 | 65 | 32,9 | 18 | 1.80 | U |

| **Additional file 2 (*continued*)** | | | | | | | | | | | |
| --- | --- | --- | --- | --- | --- | --- | --- | --- | --- | --- | --- |
| Name of proteins identified in N305 ^a^ | Spot ^b^ | *p*-value ^c^ | Fold ^d^ | Locus ^e^ | Access. ^f^ | log(E value) ^g^ | Cover. ^h^ | Mass ^i^ | NP ^j^ | PAI ^k^ | Loc. ^l^ |
| **Lipid transport and metabolism** | | | | | | | | | | | |
| 3-hydroxy-3-methylglutaryl CoA synthase | 34 | 4.15E-06 | 4.1 | mvaS | J1EX99 | -26.02 | 30 | 40.9 | 9 | 0.60 | C |
| **Inorganic ion transport and metabolism** | | | | | | | | | | | |
| Superoxide dismutase | 51, 52 | 2.44E-04 | 2.4 | sodM | J0UM22 | -18,62 | 38 | 22.9 | 5 | 0.56 | S |
| **POORLY CHARACTERIZED** | | | | | | | | | | | |
| Alpha-hemolysin | 39, 40, 41, 47 | 4.21E-07 | 6.6 | hla | J0KWK7 | -81.73 | 70 | 36.2 | 19 | 1.29 | S |
| Gamma-hemolysin component C | 40 | 4.21E-07 | 6.6 | hlgC | J1ET20 | -61.41 | 46 | 35.5 | 17 | 1.44 | S |
| Superantigen-like protein | 48 | 1.07E-04 | 3.2 | set11 | J1EYV1 | -20.88 | 28 | 25.7 | 5 | 0.50 | S |
| Superantigen-like protein 7 | 46 | 1.32E-05 | 3.2 | set5 | J1EYV6 | -58.90 | 59 | 26.0 | 14 | 1.46 | S |

^a^ Proteins of each strain are classified in COG. Names are given according to annotation of genome sequences

^b^ Spot numbers (cf Figure 4)

^c^ The ANOVA p-value indicates the degree of confidence of each fold change, determined by the SameSpot software on 3 biological replicates and 3 technical replicates

^d^ The fold change is the rate of overexpression in one of the strains, determined by the SameSpot software

^e^ Correspond to the commonly found name of the gene

^f^ Accessions numbers are given according to references on UniProtKB [35]

^g^ Probability of critical error in the protein identification given by the X!Tandem software [36]

^h^ % of the protein covered with the identified peptides

^i^ Theoretical mass as predicted from the protein sequence

^j^ Number of identified peptides

^k^ Protein abundance index ([1])

^l^ Predicted localisation based on PSORTb software. S = Extracellular C = Cytoplasmic C/M = Cytoplasmic/Membrane W = Cell wall U = Unknown
