# Supplementary material for: Fine-tuned characterization of Staphylococcus aureus Newbould 305, a strain associated with mild and chronic mastitis in bovines
Source: Vet Res. 2014 Oct 14;45(1):106. doi: 10.1186/s13567-014-0106-7 (PMC4230361; doi:10.1186/s13567-014-0106-7)
Supplement: Additional file 5: — Proteins identified as differentially expressed in the total lysate by S. aureus RF122 and N305. This table presents the proteins identified by mass spectrometry (see Materials and methods for details) in the whole cell lysates of RF122 and N305 strains. [file 13567_2014_106_MOESM5_ESM.docx]

**Additional file 3 Proteins identified as differentially expressed in the total lysate by *S. aureus* RF122 and N305.**

| Name of proteins identified in RF122^a^ | Spot^b^ | *p*-value^c^ | Fold^d^ | Locus^e^ | Access.^f^ | log(E value)^g^ | Cover.^h^ | Mass^i^ | NP^j^ | PAI^k^ | Loc.^l^ |
| --- | --- | --- | --- | --- | --- | --- | --- | --- | --- | --- | --- |
| **CELLULAR PROCESSES AND SIGNALING** | | | | | | | | | | | |
| **Post-translational modification, protein turnover, and chaperones** | | | | | | | | | | | |
| Chaperone protein DnaK | 57, 62, 63, 64, 65 | 6.30E-06 | 4.3 | dnaK | Q2YT47 | -56.49 | 33 | 66.2 | 15 | 1.00 | C |
| Alkyl hydroperoxide reductase subunit F | 57, 58, 59 | 5.01E-05 | 4.6 | ahpF | Q2YVH8 | -63.11 | 35 | 54.5 | 11 | 1.42 | C/M |
| Alkyl hydroperoxide reductase subunit C | 56 | 7.40E-04 | 2.5 | ahpC | Q2YVK2 | -43.00 | 65 | 20.9 | 9 | 1.80 | C |
| Probable protease (Zinc) protein | 53 | 3.76E-05 | 2 | SAB1141 | Q2YXN7 | -7.79 | 5 | 49.9 | 2 | 0.13 | U |
| **INFORMATION STORAGE AND PROCESSING** | | | | | | | | | | | |
| **Translation, ribosomal structure and biogenesis** | | | | | | | | | | | |
| Elongation factor Ts | 55 | 8.29E-04 | 3.7 | tsf |  |  |  |  |  |  |  |
| **METABOLISM** | | | | | | | | | | | |
| **Energy production and conversion** | | | | | | | | | | | |
| Phosphotransacetylase | 56 | 7.40E-04 | 2.5 | pta | Q2YS76 | -43.29 | 42 | 34.9 | 9 | 0.83 | U |
| ATP synthase subunit beta | 53 | 3.76E-05 | 2 | atpD | Q2YUK1 | -115.92 | 56 | 51.3 | 21 | 1.33 | C/M |
| **Carbohydrate transport and metabolism** | | | | | | | | | | | |
| Enolase | 53 | 3.76E-05 | 2 | eno | Q2YSE8 | -86.66 | 52 | 47.0 | 15 | 1.20 | C |
| Fructose-disphosphate aldolase class 1 | 60, 61 | 4.33E-04 | 6.3 | fda | Q2YWF3 | -58.15 | 55 | 32.8 | 13 | 1.40 | U |
| **Coenzyme transport and metabolism** | | | | | | | | | | | |
| Coenzyme A disulfide reductase | 54 | 2.19E-09 | 16 | cdr | Q2YWW1 | -67.61 | 29 | 49.2 | 8 | 0.42 | C |
| Name of proteins identified in N305^a^ | Spot^b^ | *p*-value^c^ | Fold^d^ | Locus^e^ | Access. ^f^ | log(E value) ^g^ | Cover.^h^ | Mass^i^ | NP^j^ | PAI^k^ | Loc.^l^ |
| **CELLULAR PROCESSES AND SIGNALING** |  |  |  |  |  |  |  |  |  |  |  |
| **Post-translational modification, protein turnover, and chaperones** | | | | | | | | | | | |
| Thioredoxin reductase | 85 | 6.52E-07 | 2.4 | trxB | J1EWC4 | -45.94 | 42 | 33.5 | 11 | 1.10 | C |

| **Additional file 3 (*continued*)** | | | | | | | | | | | |
| --- | --- | --- | --- | --- | --- | --- | --- | --- | --- | --- | --- |
| Name of proteins identified in N305^a^ | Spot^b^ | *p*-value^c^ | Fold^d^ | Locus^e^ | Access.^f^ | log(E value)^g^ | Cover.^h^ | Mass^i^ | NP^j^ | PAI^k^ | Loc.^l^ |
| Protein GrpE | 88 | 9.35E-08 | 2.8 | grpE | J0KVU6 | -7.36 | 13 | 23.9 | 2 | 0.25 | C |
| Trigger factor | 86 | 6.05E-05 | 4.4 | tig | J0KVL9 | -32.61 | 16 | 48.4 | 5 | 0.46 | C |
| **Defense mechanisms** | | | | | | | | | | | |
| Alkaline shock protein 23 | 98 | 3.80E-04 | 9.8 | asp23 | J0KY19 | -7.51 | 15 | 18.6 | 2 | 0.40 | U |
| **INFORMATION STORAGE AND PROCESSING** | | | | | | | | | | | |
| **Translation, ribosomal structure and biogenesis** | | | | | | | | | | | |
| Translation initiation factor IF-2 | 66 | 9.03E-04 | 2 | infB | J1ETX7 | -18.74 | 6 | 77.7 | 3 | 0.10 | C |
| Elongation factor Tu | 87, 93 | 1.92E-05 | 3 | tuf | J1EVM7 | -21.55 | 12 | 43.0 | 3 | 0.35 | C |
| **Replication, recombination and repair** | | | | | | | | | | | |
| DNA polymerase III subunit beta | 81 | 5.46E-05 | 3.4 | dnaN | J1EXP8 | -10.55 | 8 | 41.8 | 2 | 0.10 | C |
| **METABOLISM** | | | | | | | | | | | |
| **Energy production and conversion** | | | | | | | | | | | |
| L-lactate dehydrogenase | 83 | 1.50E-04 | 4 | ldh | J1EXA2 | -68.53 | 49 | 34.3 | 11 | 1.38 | C |
| Phosphotransacetylase | 81 | 5.46E-05 | 3.4 | eutD | J0UJS8 | -49.54 | 45 | 34.8 | 10 | 0.92 | C |
| L-lactate dehydrogenase | 74, 84 | 8.36E-07 | 5.7 | ldh | J0UMC4 | -64.29 | 42 | 34.5 | 21 | 1.75 | C |
| **Amino acid transport and metabolism** | | | | | | | | | | | |
| Ornithine cyclodeaminase protein | 77 | 5.41E-04 | 4.1 | Newbould305_0656 | J0KU07 | -5.02 | 8 | 37.7 | 2 | 0.17 | C |
| Branched-chain-amino-acid aminotransferase | 76 | 6.62E-04 | 3.4 | Newbould305_1079 | J0UJN7 | -51.83 | 47 | 40.0 | 10 | 0.78 | U |
| Branched-chain alpha-keto acid dehydrogenase subunit E2 | 67 | 1.99E-02 | 3.7 | pdhC | J0KW19 | -13.81 | 11 | 46.4 | 2 | 0.13 | C |
| D-alanine aminotransferase | 89, 91 | 3.62E-05 | 2.6 | dat | J1EZD0 | -40.52 | 41 | 31.7 | 8 | 0.59 | C |
| Proline dipeptidase | 78, 80 | 8.49E-07 | 8.2 | Newbould305_2310 | J0UJ09 | -19.86 | 20 | 39.2 | 5 | 0.50 | C |
| Oligoendopeptidase F | 66 | 9.03E-04 | 2 | Newbould305_2157 | J1EUN3 | -29.36 | 12 | 69.1 | 5 | 0.24 | C |
| Ornithine carbamoyltransferase | 78 | 8.49E-07 | 8.2 | arcB | J0ULD6 | -76.37 | 48 | 37.7 | 14 | 1.36 | C |
| **Carbohydrate transport and metabolism** | | | | | | | | | | | |
| Triosephosphate isomerase | 94 | 6.99E-04 | 2.9 | tpiA | J0UKG7 | -20.62 | 24 | 27.2 | 4 | 0.29 | C |
| Glyceraldehyde-3-phosphate dehydrogenase | 89 | 1.00E-03 | 4.7 | gap | J0KSH5 | -14.29 | 8 | 36.2 | 2 | 0.31 | C |
| **Additional file 3 (*continued*)** | | | | | | | | | | | |
| Name of proteins identified in N305 ^a^ | Spot ^b^ | *p*-value^c^ | Fold^d^ | Locus^e^ | Access.^f^ | log(E value)^g^ | Cover.^h^ | Mass^i^ | NP^j^ | PAI^k^ | Loc.^l^ |
| Phosphoglycerate kinase | 79 | 4.35E-05 | 4.4 | pgk | J1EWC9 | -63.97 | 44 | 42.5 | 11 | 0.67 | C |
| Transketolase | 67, 89 | 1.00E-03 | 4.7 | Newbould305_2114 | J1EUC2 | -84.78 | 35 | 72.1 | 16 | 0.69 | U |
| Putative translaldolase | 97 | 1.98E-04 | 2.5 | Newbould305_0104 | J1EZA8 | -24.29 | 36 | 25.6 | 6 | 0.58 | C |
| 6-phosphogluconate dehydrogenase, decarboxylating | 71 | 3.51E-05 | 3.1 | gnd | J0UIZ1 | -20.99 | 10 | 51.7 | 4 | 0.26 | C |
| **Secondary metabolites biosynthesis, transport, and catabolism** | | | | | | | | | | | |
| Acetoin reductase | 95 | 1.07E-04 | 4.1 | Newbould305_0667 | J1EXV9 | -10.20 | 16 | 27.1 | 3 | 0.38 | C |
| **POORLY CHARACTERIZED** | | | | | | | | | | | |
| UPF0447 protein Newbould305_1113 | 96 | 1.07E-04 | 2.2 | Newbould305_1113 | J1EVS0 | -16.53 | 21 | 29.3 | 4 | 0.56 | U |
| Molecular chaperone Hsp31 and glyoxalase 3 | 89, 90, 91 | 3.62E-05 | 2.6 | hchA | J1EVT7 | -32.69 | 38 | 32.0 | 8 | 1.36 | C |

^a^ Proteins of each strain are classified in COG. Names are given according to annotation of genome sequences

^b^ Spot numbers (Figure 4)

^c^ The ANOVA p-value indicates the degree of confidence of each fold change, determined by the SameSpot software on 3 biological replicates and 3 technical replicates

^d^ The fold change is the rate of overexpression in one of the strains, determined by the SameSpot software

^e^ Correspond to the commonly found name of the gene

^f^ Accessions numbers are given according to references on UniProtKB [35].

^g^ Probability of critical error in the protein identification given by the X!Tandem software [36].

^h^ % of the protein covered with the identified peptides

^i^ Theoretical mass as predicted from the protein sequence

^j^ Number of identified peptides

^k^ Protein abundance index ([1])

^l^ Predicted localisation based on PSORTb software. S = Extracellular C = Cytoplasmic C/M = Cytoplasmic/Membrane W = Cell wall U = Unknown
